# Virulence factors of *Salmonella* spp. isolated from free‐living grass snakes *Natrix natrix*

**DOI:** 10.1111/1758-2229.13287

**Published:** 2024-07-08

**Authors:** Aleksandra Pawlak, Michał Małaszczuk, Mateusz Dróżdż, Stanisław Bury, Maciej Kuczkowski, Katarzyna Morka, Gabriela Cieniuch, Agnieszka Korzeniowska‐Kowal, Anna Wzorek, Kamila Korzekwa, Alina Wieliczko, Mariusz Cichoń, Andrzej Gamian, Gabriela Bugla‐Płoskońska

**Affiliations:** ^1^ Department of Microbiology, Faculty of Biological Sciences University of Wrocław Wrocław Poland; ^2^ Laboratory of RNA Biochemistry, Institute of Chemistry and Biochemistry Freie Universität Berlin Berlin Germany; ^3^ Department of Comparative Anatomy, Institute of Zoology and Biomedical Research Jagiellonian University Kraków Poland; ^4^ Department of Epizootiology and Clinic of Birds and Exotic Animals, The Faculty of Veterinary Medicine Wrocław University of Environmental and Life Sciences Wrocław Poland; ^5^ Department of Food Hygiene and Consumer Health Protection Wrocław University of Environmental and Life Sciences Wrocław Poland; ^6^ Department of Immunology of Infectious Diseases, Hirszfeld Institute of Immunology and Experimental Therapy Polish Academy of Sciences Wrocław Poland; ^7^ Institute of Environmental Sciences Jagiellonian University Kraków Poland

## Abstract

Salmonellosis associated with reptiles is a well‐researched topic, particularly in China and the United States, but it occurs less frequently in Europe. The growth of the human population and changes in the environment could potentially increase the interaction between humans and free‐living reptiles, which are an unidentified source of *Salmonella* species. In this study, we sought to explore this issue by comparing the microbiota of free‐living European grass snakes, scientifically known as *Natrix natrix*, with that of captive banded water snakes, or *Nerodia fasciata*. We were able to isolate 27 strains of *Salmonella* species from cloacal swabs of 59 *N. natrix* and 3 strains from 10 *N. fasciata*. Our findings revealed that free‐living snakes can carry strains of *Salmonella* species that are resistant to normal human serum (NHS). In contrast, all the *Salmonella* species strains isolated from *N. fasciata* were sensitive to the action of the NHS, further supporting our findings. We identified two serovars from *N. natrix*: *Salmonella enterica* subspecies *diarizonae* and *S. enterica* subspecies *houtenae*. Additionally, we identified three different virulotypes (VT) with *invA*, *sipB*, *prgH*, *orgA*, *tolC*, *iroN*, *sitC*, *sifA*, *sopB*, *spiA*, *cdtB* and *msgA* genes, and β‐galactosidase synthesised by 23 serovars. The identification of *Salmonella* species in terms of their VT is a relatively unknown aspect of their pathology. This can be specific to the serovar and pathovar and could be a result of adaptation to a new host or environment.

## Graphical abstract

Our study reveals that free‐living snakes can carry strains of *Salmonella* species that are resistant to human serum. A comprehensive analysis of the virulence factors of the collected strains, using different methods, identified three distinct virulotypes. These are likely the result of adaptation to a new host or environment.

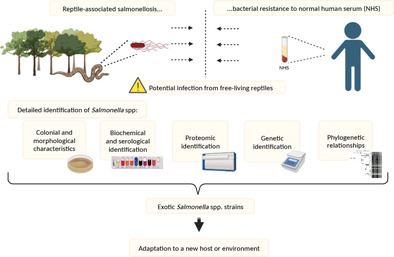

## INTRODUCTION

Zoonoses constitute a major problem for public health. The World Health Organization (WHO) estimated that 60% of emerging infectious diseases have a zoonotic origin (WHO, 2021). Since January 2020, the world has struggled with the severe acute respiratory syndrome coronavirus 2 (SARS‐CoV‐2) pandemic, which has a zoonotic origin (Holmes et al., 2021). This example also shows that zoonotic pathogens may lead to unexpected and barely controllable negative consequences for public health. Expanding knowledge about those pathogens, especially their virulence factors, is crucial for developing effective strategies to combat them. Therefore, even before the coronavirus disease 2019 (COVID‐19) pandemic, the Centers for Disease Control and Prevention (CDC) created One Health, which is an approach focusing on zoonotic diseases as a global threat (CDC, 2018). One of the most widely known bacterial zoonotic pathogens, also pointed out by One Health, is *Salmonella* spp., a causative agent of foodborne diseases and gastrointestinal infections. In 2018, *Salmonella* spp. was the second most common causative agent of gastrointestinal infections in the European Union (EU), affecting 91,857 people (European Food Safety Authority [EFSA], 2019). *Salmonella* spp. rods transmitted to humans from animals are pathogenic to people—causing non‐typhoidal salmonellosis (NTS). NTS is a global health problem, as annually worldwide, there are 1.3 billion cases of *Salmonella* spp. gastroenteritis, leading to 3 million deaths (Kurtz et al., 2017). There is still no vaccine for people, so examining transmission sources is essential to determine the ways of preventing salmonellosis. NTS is usually caused by oral transmission of *Salmonella* spp. by food and/or water but also by contact with animals.

Many animals can be vectors of salmonellosis, affecting people worldwide. For instance, in Europe, *Salmonella* spp. presence in the food chain mainly refers to eggs and poultry. However, a growing body of data indicates that one of the most essential vertebrates playing a role in spreading human salmonellosis is likely to be reptiles, known as asymptomatic carriers of *Salmonella* spp. rods, with a prevalence ranging up to 90% of reptile specimens. To date, many studies have identified the presence of more than one *Salmonella* spp. serovar in the reptile's gastrointestinal tracts (Corrente et al., 2004; Ebani et al., 2005; Geue & Löschner, 2002; Jang et al., 2008; Nakadai et al., 2005). Reptile‐associated salmonellosis (RAS) occurs mainly in children under 5 years, elderly people, and immunocompromised patients. Approximately 15% of children's RAS cases are invasive (Zając et al., 2016). *Salmonella* spp. can penetrate and colonise their intestinal tract, leading to symptoms such as diarrhoea, abdominal cramps, fever, and vomiting. Several reports from the United States and other countries described RAS cases and their origin, identifying examples of both direct and indirect contact with reptiles (Baranzelli et al., 2017; Damborg et al., 2016; Gambino‐Shirley et al., 2018; Gavrilovici et al., 2017; Horvath et al., 2016; Kiebler et al., 2020; Suzuki et al., 2017).

When analysing the National Library of Medicine's PubMed, after searching for ‘*Salmonella* reptiles,’ we found 239 results from the last 10 years (23 February 2024). Most of these studies come from the United States and China, while in Europe, the role of *Salmonella* spp. transmission connected with reptiles in the epidemiological chain is poorly examined (35 publications, PubMed 23 February 2024). In addition, the majority of these studies were conducted on captive animals maintained in zoological gardens, home collections, and households (Alix et al., 2008). There were only five original articles, including free‐living reptiles in Europe (Krautwald‐Junghanns et al., 2013; Nowakiewicz et al., 2015; Pawlak et al., 2020; Schmidt et al., 2014; Zając et al., 2016).

RAS is well known to cause numerous gastrointestinal infections in the United States, but in many European countries, this problem of public health goes unnoticed and is neglected. This disproportion shows the importance of such studies on reptiles free‐living in Europe. As we still do not have sufficient reports about *Salmonella* spp. prevalence in the gastrointestinal tract of free‐living European reptiles, the present study aims to fulfill this gap by scanning a large sample of free‐living snakes. We previously studied the Gram‐negative aerobic microbiota among the free‐living grass snake *Natrix natrix* (Pawlak et al., 2020). From a total of 45 studied reptiles, *Salmonella* species were isolated in 10 of them (10/45; 22.2%). In this study, we aim to expand the knowledge about previously and now collected *Salmonella* strains (27 strains collected from 59 *N. natrix*—snakes summed up from previous and current studies). Here, we aimed at detailed recognition of the virulence factors of all the collected strains with a wide array of approaches, including biochemical analysis, proteomic and serological identification, and analysis of the phylogenetic relationships of *Salmonella* spp. virulence genotyping, antimicrobial susceptibility, and human serum susceptibility. To show the impact of the habitat of reptiles on the composition of their intestinal microflora, we also compared 27 strains from the free‐living *N. natrix* with 3 *Salmonella* spp. strains isolated from the gastrointestinal tracts of the captive‐born banded water snake *Nerodia fasciata*. While *N. fasciata* and *N. natrix* are both semiaquatic snakes and share some ecological similarities, they are phylogenetically distinct, and the presence of *N. fasciata* in Europe is solely related to herpetoculture, as this species is native to North America (Hibbitts & Fitzgerald, 2005). Therefore, we put efforts into determining their similarities and differences in terms of virulence genotyping, antimicrobial and normal human serum (NHS) susceptibility profiles.

## EXPERIMENTAL PROCEDURES

### Sample collection from reptiles

We previously studied the Gram‐negative aerobic microbiota of 45 free‐living grass snakes *N. natrix* in Poland (Pawlak et al., 2020) determining that 22.2% of tested animals (*n* = 10) were carriers of *Salmonella* spp. Furthermore, we isolated *Salmonella* spp. from the other 14 free‐living *N. natrix* in Poland, so the total number of snakes was 59 (*n* = 59), among which we detected 27 *Salmonella* spp. strains. The previous study was focused on only determining the species composition of the Gram‐negative microbiota of free‐living snakes. In the present study, we focused on the detailed characteristics of *Salmonella* strains collected from free‐living *N. natrix*. The samples were collected from the cloaca of free‐living grass snakes (*N. natrix—*abbreviation: NN) in the Małopolskie Voivodeship (S Poland; vicinity of Kraków and Niepolomice Forest; *n* = 59) in summer during the active season and also from banded water snake (*N. fasciata*—abbreviation: NF) obtained from a private breeding colony and then maintained in the laboratory (*n* = 10), as described before (Pawlak et al., 2020). Since the microbiota of snakes is likely to be strongly affected by absorptive state and recent diet, we transferred snakes to the laboratory in the Institute of Environmental Sciences of the Jagiellonian University in Kraków to standardise sampling conditions. Snakes were maintained solitarily in disinfected terraria, with water provided *ad libitum*, and fed once per week. Before sampling, all snakes were fed with captive rodents to equalise the diet composition and get insight into the composition of *Enterobacterales*, which represent the core microbiota of snakes. Also, before being provided to snakes, rodents were kept at −20°C to deplete their own microbiota. Each snake was sampled at least 1 week after feeding to ensure it was in a post‐absorptive state (Skoczylas, 1970). Samples were collected by placing the sterile swab inside the cloaca of the snakes for 30 s with gentle rotational movements. A unique identifier for each isolated *Salmonella* spp. strain was assigned during sample collection. Moreover, *Salmonella* spp. strains were deposited in the Polish Collection of Microorganisms, Hirszfeld Institute of Immunology and Experimental Therapy, Polish Academy of Sciences, Wrocław, Poland.

### Ethical approval

Bacterial samples were collected in 2016, with the consent of the Regional Directorate for Environmental Protection in Kraków, no. OP‐I.6401.21.2015, PKw, no. OP‐I.6401.368.2016, PKw, and with the consent of the Local Ethical Committee—resolution no. 132/2015 of 26 May 2015 and resolution No. 73/2017 of 16 February 2017.

### *Identification of* Salmonella *spp. isolates*

As environmental and clinical strains of some bacterial species, including *Salmonella* spp., present atypical biochemical features (Kontou et al., 2007; Mourão et al., 2020; Mrozowska & Tyc, 1992), we decided to identify the bacterial strains using several identification methods. This ensures that the identification level is of the highest quality.

### *Colonial examination of* Salmonella *spp. isolates*

For colonial examination, the bacteria were cultured overnight at 37°C in nutrient agar (Biocorp) for 24 h to obtain pure, rounded, non‐pigmented and single colonies. This step was repeated three times. Following this incubation, a loop full of samples was streaked on MacConkey agar (Biocorp), Salmonella‐Shigella (SS) agar (Biocorp), Simmons agar (Biocorp), xylose lysine deoxycholate (XLD) agar (Merck) and Chromogenic agar (Fluka Analytical) for 24 h at 37°C. All media (except SS and XLD agar) were autoclaved at 120°C for 15 min at 0.5 atm. We assessed the grown colonies based on visual observation by determining their morphological (shape, colour, size and surface appearance) and biochemical (growth in selective media) features. This analysis was validated by comparing the grown colonies with standard clinical *Salmonella* spp. samples described in the literature, acting, in our case, as a positive control (*Salmonella enterica* serovar Typhimurium ATCC 14028, *S. enterica* serovar Montevideo ATCC 8387 and *S. enterica* serovar Oranienburg ATCC 9239—Heithoff et al., 2008; Kim et al., 2023; Maddocks et al., 2002; Pao et al., 2005; Svanevik & Lunestad et al., 2015). At this point, all isolates were initially classified into typical or atypical strains based on the visual characterisation of each bacterial colony grown in the shown above media.

### *Biochemical identification of* Salmonella *spp. isolates*

Biochemical identification of bacterial isolates was determined using the API 20E plate system purchased from Biomerieux, France (cat# 20‐100). The experiment was performed and analysed by following the manufacturer's instructions.

### *Serological identification of* Salmonella *spp.*

To determine the serological types of *Salmonella* spp. strains, the following antisera were used (Biomed, Poland): polyvalent HM antiserum (Lot No. 29420001) for *Salmonella* flagellar antigen, the different O group antisera: BO (Lot No. 25220001), CO (Lot No. 25321001), DO (Lot No. 25422001) and EO group (Lot No. 25520001) for the types of O‐somatic antigen, as well as the H:g,m antiserum (Lot No. 27120001) for the flagellar factor characteristic of *Salmonella* Enteritidis. Colonies of each *Salmonella* spp. strain was suspended in a drop of physiological saline to identify the rough form (due to their ability to auto‐agglutinate, which excludes them from the further course of the serotyping reaction). Strains showing negative auto‐agglutination results in physiological saline (the so‐called smooth strains) were qualified for further study. Agglutination was performed in the polyvalent HM antiserum first, which allowed us to confirm the belonging of the strains to the genus *Salmonella*, and then with the O groups antisera and H:g,m flagellar factor serum. The agglutination ability was checked by suspending the bacteria in a drop of serum on a glass slide and then distributing the bacterial mass throughout the drop volume, obtaining a thick, milky suspension. The slides were slightly rocked for up to 60 s in each agglutination test. Visible cell aggregates were considered a positive result for agglutination ability.

### *Proteomic identification of* Salmonella *spp. isolates (matrix assisted laser desorption/ionization ‐ time of flight ‐ mass spectrometry MALDI‐TOF MS)*

Pure single colonies of actively growing bacterial cultures were suspended in 300 μL of distilled water, then mixed after the addition of 900 μL of absolute ethanol. The supernatant was aspirated, and the pellet was dried at room temperature. Then 50 μL of 70% formic acid and 50 μL of acetonitrile were added and mixed by pipetting, followed by centrifugation at 13,000×*g* for 2 min. Next, 1 μL of supernatant was applied to the 384‐spot ground steel target plate (Bruker Daltonics, Bremen, Germany) and air‐dried at room temperature, followed by the addition of 1 μL of α‐cyano‐4‐hydroxycinnamic acid matrix solution (HCCA; Bruker Daltonics) and air‐dried again. *Salmonella* spp. strains were identified using a Bruker Daltonics UltrafleXtreme spectrometer under the control of flexControl software 3.4 (Bruker Daltonics). Biotyper 3.1 software (Bruker Daltonics) and a database containing 6904 entries were used for the identification. According to the manufacturer, the following score values were used: less than 1.7—identification not reliable, 1.7–2.0—probable genus identification, 2.0–2.3 secure genus identification and probable species identification, and more than 2.3—highly probable species identification.

### *Genetic identification of* Salmonella *spp. isolates*

#### DNA extraction

DNA was extracted from *Salmonella* spp. isolates using a commercially available Genomic Mini Kit (A&A Biotechnology, Poland) according to the manufacturer's protocol from an overnight (18–24 h, 37°C) culture.

#### Multiplex polymerase chain reaction reaction

To classify the tested *Salmonella* spp. strains, a multiplex polymerase chain reaction (PCR) assay was used in accordance with the recommendations described, with modifications (Lee et al., 2009). To amplify genes specific for *Salmonella* species/subspecies, a PCR mix (25 μL) was prepared: 10× DreamTaq Green buffer with 10 mM MgCl_2_ (Thermo ScientificTM) (2.5 μL), 10 mM dNTPs (0.5 μL) DreamTaq DNA polymerase (Thermo Scientific) 5U/10 (0.2 μL), isolated DNA template (2 μL), deionised water (18.3 μL) and 0.125 μL 0.1 mM of each primer pair of the six genes *STM4057*, *stn*, *invA*, *gatD*, *mdcA* and *fljB* (Table 1). The PCR assay was repeated twice to confirm the correctness of the assignment of the investigated strains to their respective patterns. The amplification conditions were set as follows: 94°C for 4 min of initial denaturation, 30 cycles of denaturation (45 s, 94°C), annealing (60 s, 72°C), extension (45 s, 72°C) and final extension (10 min, 72°C). Oligonucleotide primers and expected band patterns of each *Salmonella* species or subspecies are listed in Table 1.

**TABLE 1:** Oligonucleotide primers and expected band patterns of each *Salmonella* species or subspecies.

Target gene	Primer sequence (5′ → 3′)	Product size (bp)	Gene function	I	II	IIIa	IIIb	IV	V	VI
*STM4057*	F‐GGTGGCCTCGATGATTCCCG	137	Putative inner membrane protein	+	−	−	−	−	−	−
	R‐CCCACTTGTAGCGAGCGCCG									
*stn*	F‐CGATCCCTTTCCCGCTATC	179	Enterotoxin	+	+	+	+	+	+	−
	R‐GGCGAATGAGACGCTTAAG									
*invA*	F‐ACAGTGCTCGTTTACGACCTGAAT	244	Invasion protein	+	+	+	+	+	+	+
	R‐AGACGACTGGTACTGATCGATAAT									
*gatD*	F‐GGCGCCATTATTATCCTATTTAC	501	Galactitol‐1‐phosphate dehydrogenase	+	+	−	−	−	d	+
	R‐CATTTCCCGGCTATTACAGGTAT									
*mdcA*	F‐GGATGTACTCTTCCATCCCCAGT	728	Putative malonate decarboxylase	−	+	+	+	−	−	−
	R‐CGTAGCGAGCATCTGGATATCTTT									
*fljB*	F‐GACTCCATCCAGGCTGAAATCAC	848	Phase II flagellin	d	d	−	+	−	+	+
	R‐CGGCTTTGCTGGCATTGTAG									

*Note*: +, PCR product of expected size; −, no PCR product; d, differs among strains. I—*Salmonella enterica*, II—*Salmonella*. *salamae*, IIIa—*Salmonella arizonae*, IIIb—*Salmonella diarizonae*, IV—*Salmonella houtenae*, V—*Salmonella bongori*, VI—*Salmonella indica*.

### *Analysis of genetic similarity between isolated* Salmonella *spp. strains*

#### Enterobacterial repetitive intergenic consensus ERIC‐PCR

To screen genes, allowing us to discriminate the genetic similarities or differences among isolated *Salmonella* spp. strains, we performed the ERIC‐PCR reaction. Primer pairs for ERIC‐PCR amplification were as follows: ERIC‐F (5′‐AAGTAAGTGACTGGGGTGAGCG‐3′) and ERIC‐R (5′‐ATGTAAGCTCCTGGGGATTCAC‐3′). PCR mixture consisted of 10× DreamTaq Green buffer with 10 mM MgCl_2_ (Thermo ScientificTM) (2.5 μL), 10 mM dNTPs (0.5 μL), DreamTaq DNA polymerase (Thermo Scientific) 5U/10 (0.2 μL), DNA template (2 μL), deionised water (18.3 μL) and 0.125 μL of each 0.1 mM primer. The conditions of the PCR reaction were the same as for the multiplex‐PCR reaction, as shown above. The assay was repeated twice to confirm the correctness of the assignment of the investigated strains to their respective patterns.

#### Virulence genotyping

Strains were subjected to the testing of 17 virulence genes (VG) related to the pathogenicity of *Salmonella* spp. The VG genes were targeted by three following multiplex‐PCR reactions (I: *invA*, *sipB*, *prgH*, *spaN*, *orgA* and *tolC* genes; II: *iroN*, *sitC*, *lpfC*, *sifA*, *sopB* and *pefA* genes; and III: *spvB*, *spiA*, *pagC*, *cdtB* and *msgA*) performed according to Skyberg et al. (2006). The list of the primers used in this study (Genomed, Poland) and VGs functions is presented in Table 2. The amplification conditions for all three reactions were the same and set as follows: 95°C for 5 min of initial denaturation, 34 cycles of denaturation (40 s, 94°C), annealing (60 s, 66.5°C), extension (1.5 min, 72.5°C) and final extension (10 min, 72°C). PCR amplifications of each type of reaction were performed with a DNA Thermal Cycler T100 (Bio‐Rad, USA).

**TABLE 2:** Virulence genes functions and primers used in polymerase chain reaction according to Skyberg et al. (2006).

Gene	Gene function	Sequence (5′ → 3′)	Product size (bp)
*invA*	Invasion protein, TTSS formation	F:CTGGCGGTGGGTTTTGTTGTCTTCTCTATT R‐AGTTTCTCCCCCTCTTCATGCGTTACCC	1070
*sipB*	Entry into host cells	F‐GGACGCCGCCCGGGAAAAACTCTC R:ACACTCCCGTCGCCGCCTTCACAA	875
*prgH*	Invasion protein	F‐GCCCGAGCAGCCTGAGAAGTTAGAAA R‐TGAAATGAGCGCCCCTTGAGCCAGTC	756
*spaN*	Entry into host cells	F‐AAAAGCCGTGGAATCCGTTAGTGAAGT R‐CAGCGCTGGGGATTACCGTTTTG	504
*orgA*	Invasion protein	F‐TTTTTGGCAATGCATCAGGGAACA RGGCGAAAGCGGGGACGGTATT	255
*tolC*	Efflux transmembrane transporter	F‐TACCCAGGCGCAAAAAGAGGCTATC R‐CCGCGTTATCCAGGTTGTTGC	161
*iroN*	Iron acquisition	F‐ACTGGCACGGCTCGCTGTCGCTCTAT R‐CGCTTTACCGCCGTTCTGCCACTGC	1205
*sitC*	Iron acquisition	F‐CAGTATATGCTCAACGCGATGTGGGTCTCC R‐CGGGGCGAAAATAAAGGCTGTGATGAAC	768
*lpfC*	Export and assembly of fimbrial subunits	F‐GCCCCGCCTGAAGCCTGTGTTGC R‐AGGTCGCCGCTGTTTGAGGTTGGATA	641
*sifA*	Formation of Sif	F‐TTTGCCGAACGCGCCCCCACACG R‐GTTGCCTTTTCTTGCGCTTTCCACCCATCT	449
*sopB*	Maintenance of SCV	F‐CGGACCGGCCAGCAACAAAACAAGAAGAAG R‐TAGTGATGCCCGTTATGCGTGAGTGTATT	220
*pefA*	Cell adhesion	F‐GCGCCGCTCAGCCGAACCAG R‐GCAGCAGAAGCCCAGGAAACAGTG	157
*spvB*	Actin depolymerization, toxin activity	F‐CTATCAGCCCCGCACGGAGAGCAGTTTTTA R‐GGAGGAGGCGGTGGCGGTGGCATCATA	717
*spiA*	TTSS formation	F‐CCAGGGGTCGTTAGTGTATTGCGTGAGATG R‐CGCGTAACAAAGAACCCGTAGTGATGGATT	550
*pagC*	Survival within macrophages	F‐CGCCTTTTCCGTGGGGTATGC R‐GAAGCCGTTTATTTTTGTAGAGGAGATGTT	454
*cdtB*	DNA damage	F‐ACAACTGTCGCATCTCGCCCCGTCATT R‐CAATTTGCGTGGGTTCTGTAGGTGCGAGT	268
*msgA*	Survival within macrophages	F‐GCCAGGCGCACGCGAAATCATCC R‐GCGACCAGCCACATATCAGCCTCTTCAAAC	189

Abbreviations: SCV, *Salmonella*‐containing vacuole; TTSS, type III secretion system.

### Gel electrophoresis, visualisation and analysis of PCR amplification products

Amplicons were separated using a 2% agarose gel containing 4 μL of Midori Green (Nippon Genetics) at a constant voltage of 110 V for approximately 60 min using a Consort power supply. The mass marker ranged from 100 to 1000 bp (A&A Biotechnology, Cat # 3000‐500). The amplified products were visualised with Midori Green DNA (Nippon Genetics, Germany) under UV light using a Gel Doc camera system (Bio‐Rad, USA) and analysed with Quantity One software (Bio‐Rad, USA). The dendrogram was generated using BioNumerics 7.6.2 (Applied Maths) by Dice coefficient with optimisation of 0.5% and unweighted pair group method with arithmetic mean (UPGMA) clustering with a tolerance of 1.0%. The discriminatory power was calculated with Simpson's index of diversity (Hunter & Gaston, 1998).

### Serum

To determine the sensitivity of *Salmonella* spp. strains isolated from snakes to bactericidal serum activity, we purchased the pooled blood human serum (NHS) from the Regional Center for Blood Donation and Blood Treatment (Wroclaw, Poland)—arrangement No. 2039/12/2018 and No. annexe 0242/02/2020. The serum material was collected under the applicable rules included in the Act on the Public Blood Service of May 20, 2016, and in Directive 2002/980/EC of the European Parliament and the Council of 27 January 2003.

### Normal human serum bactericidal activity

The bactericidal activity of 50% NHS was determined according to the assay as previously described (Pawlak et al., 2017). Strains isolated both from *N. natrix* (II 4.1S, 39.1K, 28.1K, NN 1.3 and NN 13.3) and *N. fasciata* (NF 9.2, NF 9.4 and NF 9.5) were selected for this test. Overnight culture (5 mL of Luria–Bertani [LB] medium) was transferred (150 μL) to fresh LB medium and incubated again about 1 h to cell density equal to 0.5 in McFarland standard. Cells were centrifuged (4000 rpm, 30 min, 4°C) and suspended in 3 mL of physiological saline and then 1 mL of this was transferred to 5 mL of fresh physiological saline and mixed with NHS in one‐to‐one proportion. Each strain was incubated with 50% NHS in a water bath (37°C, 180 min). Samples were collected at the beginning of the incubation (0 min, *T*_0_) and after incubation (180 min, *T*_3_), diluted (10^1^–10^6^) and distributed (100 μL) on a solid nutrient agar medium (Biomaxima, Poland) by glass cell spreader. Plates were incubated overnight (37°C). The average number of *Salmonella* spp. colony‐forming units (CFU/mL) was estimated from three tests. To confirm the NHS bactericidal activity, the assay was performed with decomplemented (inactivated by heating, 56°C, 30 min) inactivated human serum (IHS) as the control.

### Antimicrobial susceptibility

Antimicrobial susceptibility was determined by Kirby–Bauer disc diffusion test, using Mueller–Hinton 2 agar (Biocorp) according to European Committee on Antimicrobial Susceptibility Testing guidelines (EUCAST) with the following commercial antibiotic discs (Argenta): ampicillin (Amp, 10 μg), amoxicillin/clavulanic acid (Amc, 20/10 μg), cefotaxime (Ctx, 5 μg), cefuroxime (Cxm, 30 μg), ceftazidime (Caz, 10 μg), ertapenem (Ert, 10 μg), imipenem (Imp, 10 μg), meropenem (Mem, 10 μg), ciprofloxacin (Cip, 5 μg), levofloxacin (Lev, 10 μg), pefloxacin (Pef, 5 μg), amikacin (Ak, 30 μg), tigecycline (Tig, 15 μg) and trimethoprim/sulfamethoxazole (Sxt, 1.25/23.75 μg). Plates were incubated overnight (18 ± 2 h, 35 ± 1°C) and the strains were classified as susceptible or resistant according to EUCAST clinical breakpoints (EUCAST, 2021).

## RESULTS

### *Prevalence of* Salmonella *spp. in free‐living* N. natrix

From 59 tested *N. natrix* and 10 tested *N. fasciata* individuals, 27 (45.8%) and 3 (30%) of them were carriers of *Salmonella* spp.

### *Colonial and morphological characteristics of* Salmonella *spp. isolates*

Including all 30 isolates from studied reptiles (*n* = 30/69), we observed bacterial growth on different standard selective microbial media. On Simmons citrate agar, all isolates show blue colonies, indicating their ability to assimilate citrate as the only carbon source. Furthermore, they appear yellow‐red on XLD agar, suggesting the ability to decarboxylate lysine and ferment xylose. Therefore, regarding the following biochemical features, such as the ability to (1) assimilate citrate and (2) decarboxylate lysine and ferment xylose, all isolates suggest the initial observation of typical *Salmonella* spp. (Table S1). However, on ChromAgar medium, 26/30, 87% isolates show mauve colonies, indicating their ability to produce esterase catalysing the hydrolysis of organic acid's esters, which is the feature of clinical *Salmonella* spp. samples (Heithoff et al., 2008; Kim et al., 2023; Maddocks et al., 2002; Pao et al., 2005; Svanevik & Lunestad, 2015). From a total of 30 isolates, 4 of them seem to be biochemically atypical, potentially environmental *Salmonella* spp. isolates, 13%—NN 12.2, II NN 6.1, 4.1S and II 4.1S. At the same time, 18/30 isolates appeared as non‐pigmented, rounded colonies on MacConkey media, resembling *Salmonella* spp. and sharing biochemical characteristics similar to clinical samples of *Salmonella* spp. The other 12 isolates produce pink colonies (NN 1.1, NN 1.2, NN 1.3, NN 8.1, NN 9.2, NN 11.1, NN 14.3, III NN 14.3, III NN 14.5, III NN 14.6, 28.1S and 3.1L), suggesting the ability to utilise lactose. Furthermore, we noticed transparent colonies with black centres of 20 isolates (20/30, 67%) on SS medium (H_2_S producer) suggesting the growth of *Salmonella* sp. Thus, the other 10 isolates (10/30, 33%—NN 1.1, NN 1.2, II NN 4.7, III NN 14.3, 4.1S, 7.1S, 11S, 13.3S, II 16.2K and II 4.1S) do not produce H_2_S, what is atypical feature for clinical *Salmonella* spp. strains (Table 3). The identification of all (typical—consistent with clinical samples and atypical—inconsistent with clinical samples) *Salmonella* spp. isolated from *N. natrix* (*n* = 27) and *N. fasciata* (*n* = 3) is shown in Table S1.

**TABLE 3:** The juxtaposition of all experiments from this study allowing the identification of atypical Salmonella spp exhibiting different biochemical features than clinical reference *Salmonella* spp. strains. All samples isolated from *Natrix natrix* (*n* = 27) and *Nerodia fasciata* (*n* = 3) were analysed to identify *Salmonella* spp. The identification was performed by the observation of grown colonies in selective media, followed by API 20E tests and then, MALDI‐TOF MS. All isolates prove the identification of *Salmonella* spp. Depending on which analysis is taken into account, we can detect different percentage of atypical *Salmonella* spp. strains. Note all samples should be considered to control samples, which are *Salmonella* reference strains isolated from human feaces. For the identification of all *Salmonella* spp. we refer the reader to Table S1.

Isolate	Culture media			API 20E	MALDI‐TOF MS
	SS agar^a^	MacConkey agar^b^	ChromAgar^c^	Test ONPG^d^	Score 1
Clinical sample	+	+	+	+	
NF 9.2	+	+	+	−	2.218
NF 9.4	+	+	+	−	2.199
NF 9.5	+	+	+	−	2.205
NN 1.1	−	−	+	−	2.058
NN 1.2	−	−	+	−	2.352
NN 1.3	+	−	+	−	2.203
NN 8.1	+	−	+	−	2.347
NN 9.2	+	−	+	−	2.387
NN 11.1	+	−	+	−	2.227
NN 12.2	+	+	**−**	+	2.181
NN 13.1	+	+	+	−	2.134
NN 13.3	+	+	+	−	2.144
NN 14.3	+	−	+	−	2.384
NN 14.4	+	+	+	−	2.288
II NN 4.7	−	+	+	−	2.208
II NN 6.1	+	+	**−**	+	2.098
III NN 14.3	−	−	+	−	2.291
III NN 14.5	+	−	+	−	2.212
III NN14.6	+	−	+	−	2.499
39.1K	+	+	+	−	2.409
1.2S	+	+	+	+	2.226
4.1S	−	+	**−**	+	2.244
7.1S	−	+	+	−	2.299
11S	−	+	+	−	2.392
13.3S	−	+	+	−	2.051
28.1S	+	−	+	+	2.132
3.1L	+	−	+	−	2.332
24.2L	+	+	+	−	2.187
II 16.2K	−	+	+	+	2.079
II 4.1S	−	+	**−**	+	2.371

*Note*: + represents result of the growth of *Salmonella* spp. on the selective media or biochemical features based on API20E test, which are *consistent* with a control sample: clinical reference *Salmonella* spp. − represents result of the growth of *Salmonella* spp. on the selective media or biochemical features based on API20E test, which are *not consistent* with a control sample: clinical reference *Salmonella* spp.

Abbreviation: ONPG, *o*‐nitrophenyl‐β‐D‐galactopyranoside.

^a^ Based on the growth in Salmonella‐Shigella (SS) medium, we determined that 20 *Salmonella* spp. produce H_2_S (transparent colonies with black centres, shows as +, 20/30; 66.6%), which is consistent with clinical reference *Salmonella* sp. as shown by Maddocks et al. (2002), Pao et al. (2005), Svanevik and Lunestad (2015), Heithoff et al. (2008), Kim et al. (2023). Therefore, considering the growth in SS medium, 10 *Salmonella* spp. are atypical (shown as −, NN 1.1, NN 1.2, II NN 4.7, III NN 14.3, 4.1S, 7.1S, 11S, 13.3S, II 16.2K, II 4.1S—10/30; 33.%), when compared to reference strain.

^b^ Based on the growth in MacConkey medium, we determined that 18 *Salmonella* spp. do not utilise lactose (non‐pigmented, rounded colonies, shows as +, 18/30; 60%), which is consistent with clinical reference *Salmonella* species as shown in Maddocks et al. (2002), Pao et al. (2005), Svanevik and Lunestad (2015), Heithoff et al. (2008), Kim et al. (2023). Therefore, considering the growth in MacConkey medium, 12 *Salmonella* spp. are atypical (shown as −, NN 1.1, NN 1.2, NN 1.3, NN 8.1, NN 9.2, NN 11.1, NN 14.3, III NN 14.3, III NN 14.5, III NN 14.6, 28.1S, 3.1L—12/30; 40%) when compared to reference strain.

^c^ Based on the growth in ChromAgar medium, we determined that 26 *Salmonella* spp. produce esterases (mauve colonies, shown as +, 26/30, 87%), which is consistent with clinical reference *Salmonella* species as shown in Maddocks et al. (2002), Pao et al. (2005), Svanevik and Lunestad (2015), Heithoff et al. (2008), Kim et al. (2023). Therefore, considering the growth in ChromAgar medium, four *Salmonella* spp. were atypical (no mauve colonies, shown as −, NN 12.2, II NN 6.1, 4.1S and II 4.1S—4/30; 13%) when compared with reference strain.

^d^ Based on ONPG test in API20E test, we determined that seven *Salmonella* spp. were negative (no change in colour) (NN 12.2, II NN 6.1, 1.2S, 4.1S, 28.1S, II 16.2K and II 4.1S—7/30; 23.3%), which is consistent with reference *Salmonella* species as shown in Boadi et al. (2010). Therefore, considering results from ONPG test, 23 *Salmonella* spp. were atypical when compared to reference *Salmonella* spp. strains (23/30; 76.6%).

### *Biochemical and serological identification of* Salmonella *spp. isolates*

On the API 20E test, all suspected *Salmonella* spp. isolates (*n* = 30) ferment glucose and arabinose, decarboxylate lysine and ornithine, assimilate citrate, and produce H_2_S and NO_2_ (Table S1), which is consistent with clinical reference *Salmonella* sp. Furthermore, all *Salmonella* spp. are incapable of producing urea, do not break down tryptophan to indole and are devoid of cytochrome oxidase—the final enzyme of the respiratory chain in oxygen respiration (Table S1) exhibiting the same biochemical features as positive control sample: clinical reference *Salmonella* spp. In contrast, 77% of *Salmonella* spp. strains (23/30) synthesise β‐galactosidase. Moreover, *o*‐nitrophenyl‐β‐D‐galactopyranoside (ONPG), under the influence of the β‐galactosidase enzyme, is hydrolyzed to galactose and *o*‐nitrophenol, which causes the substrate to turn yellow—visual evidence of the enzyme's presence. Thus, seven isolates referred to as NN 12.2, II NN 6.1, 1.2S, 4.1S, 28.1S, II 16.2K and II 4.1S show the negative result on ONPG wells. We compared these results with the reference *Salmonella* strain isolated from the faeces of patients with salmonellosis (Heithoff et al., 2008; Kim et al., 2023; Maddocks et al., 2002; Pao et al., 2005; Svanevik & Lunestad, 2015). Considering that *Salmonella* species lack the β‐galactosidase enzyme (Boadi et al., 2010), we determined that 23 tested isolates are atypical—the ONPG test was positive (colour change) (23/30; 76,6%). The biochemical features of all *Salmonella* sp. isolated from *N. natrix* (*n* = 27) and *N. fasciata* (*n* = 3) are shown in Table S1.

All *Salmonella* isolates were agglutinated with the polyvalent flagellar HM antiserum. In opposite to HM antiserum, other reactions with O group antisera and H:g,m flagella antiserum were negative.

### *MALDI‐TOF MS identification of* Salmonella *spp. isolates*

MALDI‐TOF MS classified all bacterial samples as *Salmonella* spp. group with 98.32%–100% sequence similarity. Twenty‐one tested isolates resulted in secure genus identification (score: 2.0–2.3) and nine of the strains with highly probable species identification as *S. enterica* (score more than 2.3—NN 1.2, NN 8.1, NN 9.2, NN 14.3, III NN14.6, 39.1K, 11S and II 4.1S 3.1L) (Table 3).

### *Genetic identification of* Salmonella *spp. isolates*

**FIGURE 1:**
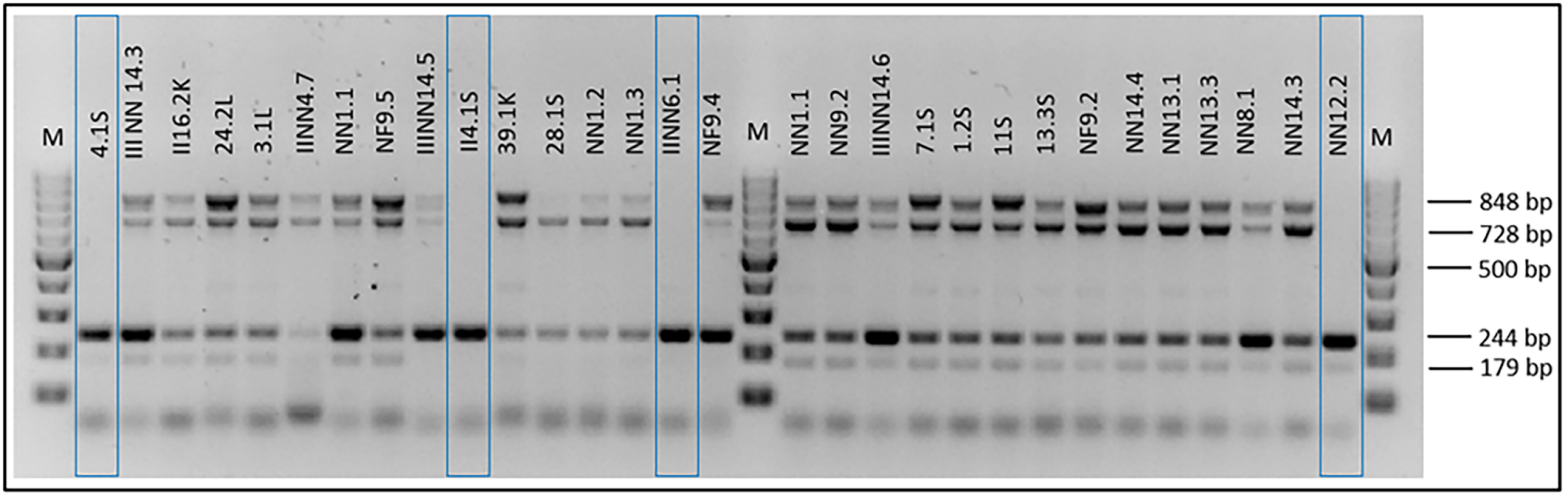
The classification of *Salmonella* isolates into different subspecies based on the detection of specific genes for each subspecies (according to Lee et al., 2009). In blue boxes we highlighted *Salmonella* spp. isolates identified as *Salmonella enterica* subsp. *houtenae* (4.1S, II4.1S, IINN.6.1 and NN12.2); M—DNA molecular weight marker.

Based on the data from a multiplex‐PCR assay with six primer pairs (*STM4057*, *stn*, *invA*, *gatD*, *mdcA* and *fljB* genes), we classified *Salmonella* isolates into different subspecies. Twenty‐six isolates were characterised by the detection of *stn*, *invA*, *mdcA* and *fljB* genes as *S. enterica* subsp. *diarizonae* (Figure 1). In contrast, the remaining four isolates (4.1S, II 4.1S, II NN6.1 and NN 12.2) were demonstrated to be *S. enterica* subsp. *houtenae* because of the detection of only two of the *stn* and *invA* genes. The *STM4057* and *gatD* genes were not detected in any of the tested samples. Table 2 represents the sizes, sequence primers and functions of each gene, allowing us to differentiate *Salmonella* sp. isolates.

### The determination of phylogenetic relationships—ERIC‐PCR

**FIGURE 2:**
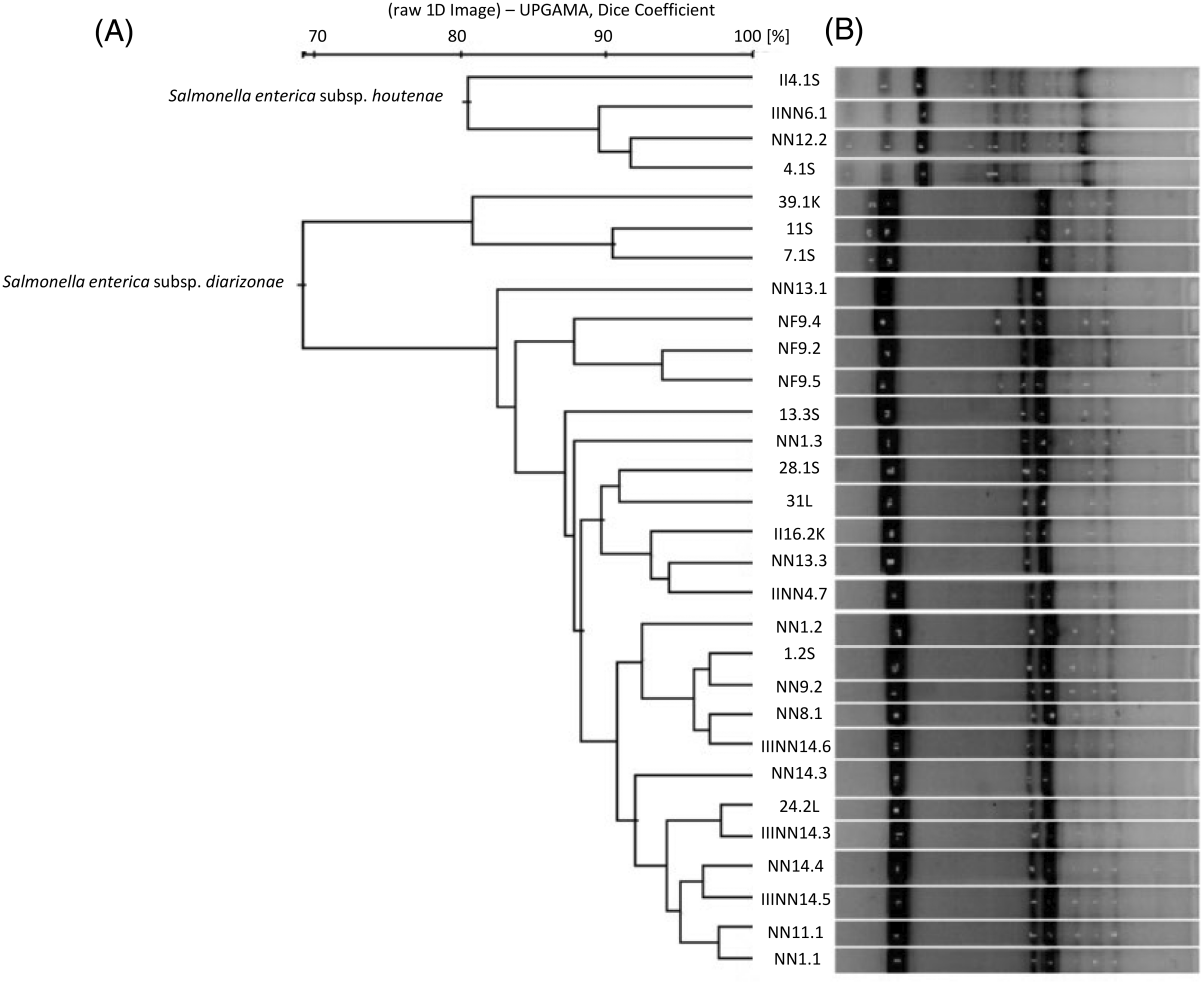
The determination of genetic correlation of *Salmonella* spp. strains isolated from reptiles based on ERIC‐PCR. (A) Agarose gel as in Figure 1 indicating strictly conserved fragments of the genome of studied *Salmonella* spp. isolates enabling the determination of the genetic similarities between them. (B) Cluster analysis of ERIC‐PCR profiles of *Salmonella* spp. with their corresponding origin. The phylogenetic tree was generated by the average linkage agglomeration method (unweighted pair group method with arithmetic mean) using BioNumerics 7.6.2 (Applied Maths).

Based on the ERIC‐PCR reaction, all *Salmonella* spp. isolates from reptiles (*n* = 30) are genetically comparable. The cluster similarity cut‐off value was set at least 69% (Figure 2). The overall Simpson's index of diversity for ERIC‐PCR clustering was 0.69. However, the previous experiment indicated the detection of four *Salmonella* spp. isolates belonging to *S. enterica* subsp. *houtenae* (4.1S, II 4.1S, NN12.2 and II NN 6.1), we confirm that these strains differ genetically from the remaining tested isolates. Therefore, according to the phylogenetic tree presented in Figure 2, we generated two main clusters, one of which belongs only to *S. enterica* subsp. *houtenae*. Comparing these isolates, the genetic similarity was referred to as approximately 80%. The second cluster included all 26 isolates of *S. enterica* subsp. *diarizonae*. We classified them into two smaller subclusters, one of which consisted of three genetically similar isolates: 39.1K, 11S and 7.1S. These three strains were distinguished from the others by the lack of one of the two products weighing over 800 bp. The second subcluster was more diverse. However, even in this case, we were able to find some strong correlations between the genetic profiles of analysed *Salmonella* spp. isolates. For instance, according to the phylogenetic tree, isolates: NN 11.1 and NN 1.1 have substantial genetic similarities (approximately 97%). It is worth noting the profiles of *Salmonella* spp. isolates from the same snake species: *N. fasciata* (NF 9.2, NF 9.4 and NF 9.5). Their relatively close localization in the phylogenetic tree (with an additional product around 700 bp) and belongingness in the same subcluster reflect their high genetic similarity. This result also confirms comparable proteomic and biochemical characteristics observed in previously described experiments (culture methods, API 20E test and MALDI‐TOF MS, Table 3).

### Virulence genotyping

All tested *Salmonella* spp. were genotyped by PCR as described (Skyberg et al., 2006). The presence of 12 out of 17 amplified genes was confirmed. The VG were categorised into three distinct virulotypes (VT): A, B and C. The full gene was the only difference compared to the A type. All of the *S. houtenae* strains had identical VT (C) and the PCR confirmed the presence of *prgH* and *sifA* gene only in this VT. All the tested *Salmonella* spp. strains contained *msgA*, *tolC*, *sitC*, *sipB* and *sopB* genes. VT A and B contained additional *invA*, *orgA* and *spiA* genes and were the most common (VT‐A in 67% and VT‐B in 20% of tested strains). The only difference between VT‐A and VT‐B was the *ironN* gene, presented in VT‐B and VT‐C, but not in VT‐A type. All of the strains with VT‐A and VT‐B were classified as *S. enterica* subsp. *diarizonae*. *SifA* and *prgH* genes were detected only in VT‐C, which was presented in each (4/4) *S. enterica* subsp. *houtenae* strains; these genes were not detected in other isolates. None of the strains contained the *spvB*, *pagC*, *lpfC*, *pefA* and *spaN* genes (Table 4).

**TABLE 4:** The taxonomic classification of all *Salmonella* spp. isolated from analysed reptiles (based on data from Lee et al., 2009) and the determination of their virulotypes (according to Skyberg et al., 2006). Note virulotypes A and B belong to *Salmonella diarizonae*, while virulotype C belongs to *Salmonella houtenae*.

	*invA*	*sipB*	*prgH*	*spaN*	*orgA*	*tolC*	*iroN*	*sitC*	*lpfC*	*sifA*	*sopB*	*pefA*	*spvB*	*spiA*	*pagC*	*cdtB*	*msgA*	VT^a^	Taxonomic classification
II 4.1S																		*C*	*S. houtenae*
II NN6.1																		*C*	*S. houtenae*
NN 12.2																		*C*	*S. houtenae*
4.1S																		*C*	*S. houtenae*
39.1K																		B	*S. diarizonae*
11S																		B	*S. diarizonae*
7.1S																		B	*S. diarizonae*
NN 13.1																		A	*S. diarizonae*
NF 9.4																		A	*S. diarizonae*
NF 9.2																		B	*S. diarizonae*
NF 9.5																		B	*S. diarizonae*
13.3S																		A	*S. diarizonae*
NN 1.3																		A	*S. diarizonae*
28.1S																		A	*S. diarizonae*
3.1L																		A	*S. diarizonae*
II 16.2K																		A	*S. diarizonae*
NN 13.3																		A	*S. diarizonae*
II NN 4.7																		A	*S. diarizonae*
NN 1.2																		A	*S. diarizonae*
1.2S																		A	*S. diarizonae*
NN 9.2																		A	*S. diarizonae*
NN 8.1																		A	*S. diarizonae*
III NN 14.6																		A	*S. diarizonae*
NN 14.3																		A	*S. diarizonae*
24.2L																		B	*S. diarizonae*
III NN 14.3																		A	*S. diarizonae*
NN 14.4																		A	*S. diarizonae*
III NN 14.5																		A	*S. diarizonae*
NN 11.1																		A	*S. diarizonae*
NN 1.1																		A	*S. diarizonae*

*Note*: Black field—positive result—detection of the expressed gene, white field—negative result—the following gene was not expressed.

Abbreviation: VT, virulotype.

^a^ Each *Salmonella* spp. isolate was categorized to different type of virulence (A—in orange, B—in blue, C—in green).

### Normal human serum activity

As mentioned before, selected *Salmonella* spp. strains isolated from *N. natrix* (*n* = 5) and *N. fasciata* (*n* = 3) were tested for bactericidal action of NHS. At the beginning of incubation with NHS, the average cell count ranged from 2.5 × 10^6^ to 4.9 × 10^6^ CFU/mL. All of the tested *Salmonella* spp. strains isolated from *N. natrix* were resistant to bactericidal action of 50% NHS, whereas all the strains isolated from *N. fasciata* were susceptible. Two strains (NN 1.3 and 28.1K) demonstrated a highly resistant level to bactericidal action of NHS and obtained 1.0 × 10^8^ and 4.3 × 10^7^ CFU/mL, respectively (Table 5). The remaining of the tested strains isolated from *N. natrix* (II 4.1S, NN 13.3 and 39.1K) demonstrated lower but still serum resistance to NHS and reached 608%, 615% and 358% of survival, respectively, after 3 h of incubation. The count of the colonies of strains isolated from *N. fasciata* (NF 9.2, NF 9.4 and NF 9.5) did not exceed the 4.8 × 10^3^ CFU/mL after 3 h of incubation with NHS, which was less than 1% of survival. All of the strains isolated from both *N. natrix* and *N. fasciata* survived after 3 h control incubation with ISH and reached cell count 2.5 × 10^7^ to 1.6 × 10^8^ CFU/mL (Table 5). The most serum‐resistant strains (NN 1.3 and 28.1K) also demonstrated the best growth in control conditions. Strains susceptible to the NHS were able to grow with IHS.

**TABLE 5:** Colony‐forming units (CFU) per millilitre and percent of survival of *Salmonella* spp. cells after 3 h incubation in 50% normal human serum (NHS) and 50% inactivated human serum (IHS).

*Salmonella* spp. strain	50% NHS		Percent of survival of colonies	50% IHS		Percent of survival of colonies
	CFU/mL			CFU/mL		
	*T*_0_	*T*_3_		*T*_0_	*T*_3_	
Strains resistant to 50% NHS						
II 4.1S	2.7 × 10^6^	1.7 × 10^7^	608	2.9 × 10^6^	2.2 × 10^7^	751
NN 1.3	4.9 × 10^6^	1.2 × 10^8^	2223	4.6 × 10^6^	1.6 × 10^8^	3511
NN 13.3	2.5 × 10^6^	1.5 × 10^7^	615	4.1 × 10^6^	4.6 × 10^7^	1113
39.1K	3.2 × 10^6^	1.1 × 10^7^	358	5.5 × 10^6^	6.2 × 10^7^	1121
28.1K	4.9 × 10^6^	4.3 × 10^7^	1305	2.0 × 10^6^	4.5 × 10^7^	2245
Strains sensitive to 50% NHS						
NF 9.2	4.3 × 10^6^	4.8 × 10^3^	<1	1.5 × 10^6^	1.2 × 10^7^	759
NF 9.4	2.6 × 10^6^	1.0 × 10^3^	<1	4.8 × 10^6^	2.3 × 10^7^	489
NF 9.5	2.8 × 10^6^	0	0	4.9 × 10^6^	2.5 × 10^7^	503

### Antimicrobial susceptibility

All the *Salmonella* spp. strains were susceptible to 13 of the14 antimicrobial agents used in this study and 80% of strains were susceptible to all tested antibiotics. Growth inhibition zones with antibiotics are presented in Table 6. Tigecycline was the only ineffective antimicrobial agent for some of the tested *Salmonella* spp. strains. All the strains resistant to tigecycline were *S. enterica* subsp. *diarizonae* isolated both from *N. natrix* (7.1S, 13.3S, NN 1.1, NN 1.2 and III NN 14.5) and *N. fasciata* (NF 9.5).

**TABLE 6:** Growth inhibition zones of *Salmonella* spp. (*n* = 30) in Kirby–Bauer disc diffusion test with the clinical breakpoints for *Enterobacterales* according to European Committee on Antimicrobial Susceptibility Testing guidelines.

Inhibition zone (mm)	Antimicrobial agent													
	Amp	Amc	Cxm	Ctx	Caz	Ert	Mem	Imp	Ak	Cip	Pef	Lev	Tig	Sxt
10	x	x	x	x	x	x	x	x	x	x	x	x	x	x
11	x	x	x	x	x	x	x	x	x	x	x	x	x	x
12	x	x	x	x	x	x	x	x	x	x	x	x	x	x
13	x	x	x	x	x	x	x	x	x	x	x	x	x	x
14	x	x	x	x	x	x	x	x	x	x	x	x	x	x
15	x	x	x	x	x	x	x	x	x	x	x	x	2	x
16	x	x	x	x	x	x	x	x	x	x	x	x	4	x
17	x	x	x	x	x	x	x	x	x	x	x	x	x	x
18	1	x	x	x	x	x	x	x	x	x	x	x	23	x
19	4	x	x	x	x	x	x	x	x	x	x	x	1	x
20	12	x	x	x	x	x	x	x	x	x	x	x	x	x
21	1	x	x	x	x	x	x	1	x	x	x	x	x	x
22	5	x	x	x	x	x	2	x	x	x	x	x	x	x
23	1	x	x	x	x	x	x	4	x	x	x	x	x	1
24	4	x	x	1	x	x	1	4	3	x	9	1	x	x
25	x	x	4	x	x	1	9	13	4	x	14	1	x	x
26	1	1	5	x	1	8	3	1	11	x	6	1	x	x
27	x	x	4	1	x	4	9	6	1	x	1	4	x	1
28	x	7	5	x	10	4	4	1	5	1	x	5	x	3
29	x	2	5	x	x	x	1	x	x	1	x	1	x	1
30	1	11	6	7	11	10	1	x	4	2	x	13	x	13
31	x	1	1	2	3	1	x	x	x	1	x	x	x	8
32	x	5	x	6	3	2	x	x	2	x	x	1	x	2
33≤	x	3	x	13	2	x	x	x	x	25	x	3	x	1

*Note*: numbers—number of detected strains with indicated inhibition zone; x—indicated inhibition zone not detected in tested strains; white field—zone for susceptible strains; grey field—zone for resistant strains.

Abbreviations: Ak, amikacin; Amc, amoxicillin/clavulanic acid; Amp, ampicillin; Caz, ceftazidime; Cip, ciprofloxacin; Ctx, cefotaxime; Cxm, cefuroxime; Ert, ertapenem; Imp, imipenem; Lev, levofloxacin; Mem, meropenem; Pef, pefloxacin; Sxt, trimethoprim/sulfamethoxazole; Tig, tigecycline.

## DISCUSSION

### Salmonella *spp. prevalence in free‐living snakes*

Our study concerning the prevalence of *Salmonella* spp. in reptiles is the first on such a large sample of free‐living snakes in Europe. We indicated that free‐living *N. natrix* are common carriers of *Salmonella* spp. This finding gives us strong evidence that *Salmonella* spp. rods are a part of the intestinal microbiota of snakes native to Poland. Another study on *N. natrix* in Poland but on a smaller number of snakes (*n* = 15) showed that 87.5% of them were *Salmonella* spp. carriers (Zając et al., 2016). The finding of *Salmonella* sp. prevalence in reptiles was also shown in our previously reported studies. Dudek et al. (2019) isolated 15 *Salmonella* spp. strains from 84 samples collected from reptiles housed in Wroclaw Zoo, Poland (15/84; 17.8%). Consistently, Pawlak et al. investigated cloacal Gram‐negative microbiota of 45 free‐living grass snakes (*N. natrix*). *Salmonella* spp. was present in 10 cloacal swabs (10/45, 22.2%) (Pawlak et al., 2020). Considering data outside of Poland, several studies focused on the analysis of *Salmonella*—prevalence in European free‐living reptiles. For instance, Hacioglu et al. studied eight free‐living *N. natrix* in Turkey and nine of the swabs contained *Salmonella arizonae* (Hacioglu et al., 2015). In contrast, studies from Germany showed that *Salmonella* spp. was not isolated from the intestinal tracts of free‐living *N. natrix* (*n* = 12). The same study showed that 8/23 (34.8%) free‐living *Vipera berus* were the carriers of *Salmonella* spp. (Schmidt et al., 2014). Furthermore, Lukać et al. found no *Salmonella* spp. isolates in the gastrointestinal tracts of free‐living four‐lined snakes *Elaphe quatuorlineata* (*n* = 20) (Lukač et al., 2017). Those limited European studies on free‐living snakes show opposite results, which may indicate a high population‐ or/and species‐specificity. A greater number of populations and species are needed to be scanned for the presence of *Salmonella* spp. to assess environmental correlates of *Salmonella* prevalence.

To date, the highest prevalence of *Salmonella* spp. was recorded in Europe in captive reptiles (Bjelland et al., 2020; Bošnjak et al., 2016; Dudek et al., 2019; Hydeskov et al., 2013; Marin et al., 2021; Pees et al., 2013; Russo et al., 2018; Wikström et al., 2014). Our study populations are located within a large city and in its close vicinity, which is associated with a high risk of contact between wild snakes and captive ones that are accidentally or deliberately released into nature. This phenomenon raises the question of whether *Salmonella* spp. occurring in free‐living versus captive populations are independent strains or if the frequent occurrence of *Salmonella* spp. in wild snakes can originate through contact with other animals naturally existing in the environment rather than captive snakes. For instance, Bauwens et al. reported the presence of *Salmonella* spp. rods outside the terrariums of captive reptiles, which indicates that the pathogenic bacteria can be easily transmitted to the environment by domestic animals and/or humans (Bauwens et al., 2006). This indicates that *Salmonella* can probably be easily transferred from captive reptiles to the environment via the activity of humans. In our study, grass snakes were collected in Craców and closely located sites. These areas are densely populated with a well‐developed herpetoculture and cases of released into the nature of alien reptiles are regularly reported. This results in an increase in the risk of contact with native species, which—together with easiness of *Salmonella* spread—raises the question of the role of captive reptiles in the distribution of *Salmonella* in the wild (Morydz et al., 2017). Reptiles can be colonised by *Salmonella* spp. at the very early stage of life through transovarial transmission, direct contact with other reptiles or with their faeces (Mermin et al., 1997). This suggests the maintenance of microbiota through generations, which increases the time window over which *Salmonella* can be transferred into the environment.

Thus, preventing the zoonotic risk associated with *Salmonella* spp. occurring in free‐living snakes should be focused on controlling captive reptiles' maintenance. In addition, an essential aspect of zoonotic control can be related to the conservation of natural populations of reptiles. This is because harvesting or deliberate persecution of free‐living species is associated with direct contact with animals, which is a risk factor for infection.

A well‐known example of snake harvesting is in Asian wet markets, including Wuhan in China, where the COVID‐19 pandemic originated. Secondly, greater densities of the host population can reduce the prevalence of particular parasites of pathogens due to the dilution effect and vice versa—a vastly decreased population number can be predicted by the locally elevated density of several pathogens and increase the risk of their further spread, for example, into humans.

There are many other anthropogenic factors increasing zoonoses spreading: poverty and culinary traditions of wild animals' meat consumption, climate change resulting in the emergence of new species in places where they did not exist before, urbanisation of the natural environment of wild animals' occurrence, an increase in the frequency and distance of human travel and international trade (Magouras et al., 2020).

### Virulence

Invasive NTS and serious NTS in patients from risk groups require antibiotic treatment, which efficiency can be reduced by drug resistance of particular *Salmonella* spp. strains (Krzyżewska‐Dudek et al., 2022; Nishio et al., 2005; Ondari et al., 2019; Rossi et al., 2019). In Europe, Poland has one of the highest levels of drug‐resistant *Salmonella* spp. strains, including multidrug‐resistant strains (MDR) and extended‐spectrum beta‐lactamases (ESBL) strains (EFSA & ECDC, 2019). In most cases, *Salmonella* spp. strains found in reptiles reveal mild to strong levels of drug resistance. In our study, we reported mildly potent drug resistance to only tigecycline in five serovars isolated from free‐living *N. natrix* and one strain isolated from captive *N. fasciata*. Tigecycline is an important drug active against many bacteria, including drug‐resistant pathogens like methicillin‐resistant *Staphylococcus aureus* (MRSA) and methicillin‐resistant *Staphylococcus epidermidis* (MRSE), vancomycin‐resistant *Enterococcus* species (VRE), extended‐spectrum‐beta‐lactamases‐producing *Enterobacteriaceae* and multi‐drug and extensively‐drug‐resistant (MDR) *Acinetobacter baumannii* (Kim et al., 2016; Townsend et al., 2007). Tigecycline is a recommended drug for the treatment of serious bacterial infections, like complicated skin and tissue infections, intra‐abdominal infections, pneumonia and osteomyelitis and is currently the last‐line drug against MDR bacterial pathogens, including carbapenem‐resistant *Enterobacteriaceae* (Yaghoubi et al., 2022). Tigecycline‐resistant *Salmonella* spp. is rarely described; however, high percentage (93.1%) of non‐susceptible (intermediate or resistant) *Salmonella* isolated from healthy reptiles have been reported in Italy (Bertelloni et al., 2016). Tigecycline‐resistant *Salmonella* spp. were also isolated from poultry production areas and humans (Wang et al., 2023, Abd El‐Aziz et al., 2021). It has been described that the activity of AcrAB–TolC efflux pump system and the presence of tigecycline‐resistant gene tet(X4) are two main mechanisms of resistance to this drug (Chetri et al., 2019; Zhang et al., 2022). We found the *tolC* gene in each *Salmonella* virulotype described in this research and further analyses will be performed. Other studies on European snakes, for example, Schmidt et al. reported resistance to streptomycin of *Salmonella* spp. strains isolated from free‐living *Vipera berus* (Schmidt et al., 2014). Research by Cota et al., similar to ours, indicates low resistance of *Salmonella* spp. isolates from reptiles to antibiotics. *Salmonella* spp. and *Salmonella* IIIb obtained from *Pantherophis guttatus guttatus* and *Python regius* were sensitive to tested fluoroquinolones, aminoglycosides, amoxicillin with clavulanic acid, and ampicillin but resistant to penicillin (Cota et al., 2021). On the other hand, *Salmonella* Kentucky isolated from snakes in Poland were resistant, among other drugs, to ampicillin and ciprofloxacin (Zając et al., 2013). It is crucial for RAS‐causing *Salmonella* to be treatable, as seen with *Salmonella* IIIb isolated in Romania, which was sensitive to ampicillin, ciprofloxacin, trimethoprim–sulfamethoxazole, and third‐generation cephalosporin (Gavrilovici et al., 2017). However, in Romania, there was already isolated resistant *Salmonella* spp., including resistance to first‐ and second‐generation cephalosporins and aminoglycosides. (Cristina et al., 2022). Marin et al. (2021) studied reptiles from households and pet shops, where 72% of strains were MDR, and most frequently they observed gentamicin‐colistin and gentamicin‐colistin‐ampicillin resistance patterns. MDR strains were also reported in Wuhan, China—the epicentre of the COVID‐19 outbreak. In 2020, Xia Y et al. isolated MDR *Salmonella* spp. from the lungs of edible snakes with pneumonia. The strain was resistant to 14 antibiotics. Moreover, the authors conducted the lethal test of isolated *Salmonella* spp. serovar in chickens, resulting in 75% mortality in chickens in 24 h (Xia et al., 2020). This example strongly underlines the zoonotic potential of *Salmonella* spp. strains isolated from snakes and its presence in food chains and the possibility of infecting people.

Besides the drug resistance, we tested the bactericidal action of 50% NHS on isolated *Salmonella* spp. strains. All strains isolated from free‐living *N. natrix* were resistant, and the strains isolated from captive *N. fasciata* were sensitive to NHS action. Given that the immunity of infants and small children is highly based on innate immune response mechanisms such as complement contained in human serum, the resistance of bacteria to NHS can be one of the factors leading to invasive infections. In our previous studies, we indicated that *Salmonella* strains even from the same serotype group vary in their susceptibility to NHS. Many strains were sensitive to the bactericidal action of serum. Multiple passages of some strains in serum led to achieving resistance to NHS (Bugla‐Płoskońska et al., 2009; Pawlak et al., 2017). In the present study, all tested strains were resistant to human serum, which suggests that the long‐term carrier of *Salmonella* strains in reptile intestines might be a factor that leads to serum resistance.

We analysed the presence of genes that are the main determinants of virulence in vertebrates (Alix et al., 2008; Dudek et al., 2019; Gunn et al., 1995; Krawiec et al., 2015; Lilic et al., 2010; Skyberg et al., 2006; Stein et al., 1996). We detected 12 of the 17 amplified genes in tested *Salmonella* spp. strains. However, essential bacterial invasion VG are missing, including *spvB*, *pagC*, *lpfC*, *pefA* and *spaN*. These genes are responsible for toxicity, cell adhesion, survival within macrophages, export and assembly of fimbrial subunits, and entry into host cells, respectively. Pasmans et al. (2005) found *pef* A only in one (from 79) *Salmonella* spp. strain isolated from captive reptiles. Bertelloni et al. (2016) detected *spv* genes (important for macrophage cytotoxicity and destabilisation of the cytoskeleton of the eukaryotic cell) also in only one (from 29) *Salmonella* spp. serovar. In our previous study, Dudek et al., aimed to determine VGs profiles for both *S. enteritidis* from humans and *Salmonella* strains from reptiles housed in a Polish zoo (Dudek et al., 2019). *Salmonella* strains from reptiles revealed lower prevalence of VG compared to *Salmonella* strains isolated from humans. In line with our study, the *sitC* gene was observed in all tested *Salmonella* strains. However, genes that were not detected in our *Salmonella‐*tested strains (Table 4) were highly expressed in *Salmonella* strains isolated from captive reptiles, including, *lpfC* (27%), *spaN* (47%) and *pagC* (80%) genes (Dudek et al., 2019). Furthermore, 93% of clinical *Salmonella* strains possessed the *invA* gene; its expression was also observed among our tested environmental strains, except those identified as *S. houtenae* (4.1S, II 4.1S, NN12.2 and II NN 6.1). Additionally, as Dudek et al., determined, gene *cdtD* was not present in clinical *Salmonella* strains, it was expressed in 40% of reptilian *Salmonella* strains. In our case, this gene was present in all *Salmonella* strains, except those belonging to *S. houtenae*. In line with Dudek et al. (2019) study, none of the tested *Salmonella* strains isolated from reptiles had *spvB* and *pefA* genes in its genome. MDR *Salmonella* spp. strain isolated by Xia et al. (2020) despite its high mortality in chickens and ability to cause pneumonia in snakes, also lacks *spv* genes. In this research, we classified the isolated strains into two subspecies: *S. enterica* subsp. *diarizonae* and *S. enterica* subsp. *houtenae*. The strains of the subspecies *diarizonae* showed phenotypic and genotypic variability, which may be the basis for further research, including the typification of surface antigens.

### Final remarks

Our study indicates that free‐living snakes in Europe can be carriers of *Salmonella* spp. strains that have poor ability to invade host cells; however, with resistance to human serum. The bacteria should be treated with clinical importance to risk groups, especially infants and very young children. So far, to our knowledge, it is the biggest study on snakes living in Europe. It is still not fully clear whether *Salmonella* spp. is a part of the natural intestinal microbiota of reptiles or an opportunistic pathogen. Most reptiles do not show any symptoms of salmonellosis; however, some factors including stress, diet change, food deprivation, exposure to cold, or transportation may develop symptoms such as diarrhoea, vomiting, anorexia, pneumonia, and sepsis, leading even to death (CDC, 2006; Ebani et al., 2005; Bjelland et al., 2020; Xia et al., 2020). It is a very interesting phenomenon, as for people *Salmonella* spp. is a virulent pathogen and transmission of *Salmonella* spp. serovars from reptiles to humans can lead to RAS. RAS cases are not monitored in Poland and are poorly monitored in some European countries, for example, Germany (Pees et al., 2013; Schneider et al., 2009), Belgium (Meervenne et al., 2009), France (Perez Costa et al., 2015; Horvath et al., 2016), the Netherlands (Mughini‐Gras et al., 2016), Norway (Bjelland et al., 2020), Portugal (Cota et al., 2021), Romania (Gavrilovici et al., 2017), and Spain (Bertrand et al., 2008; Perez Costa et al., 2015; Harris et al., 2010). On the other hand, the United States struggles with RAS and monitors the cases, noting even local outbreaks (Cain et al., 2009, Meyer Sauteur et al., 2013, Whiley et al., 2017, Centers for Disease Control and Prevention [CDC], 2020). RAS is mainly a health problem for young children, immunocompromised patients, and the elderly population. The younger the child is, the more considerable the risk of invasive salmonellosis. Although turtles are reported to be the most often source of *Salmonella* rods causing children's RAS, the invasive forms of *Salmonella* are transmitted more often from other reptiles, including lizards and snakes (Vora et al., 2012). We indicated the occurrence of VG (*invA*, *sipB*, *prgH*, *orgA*, *tolC*, *iroN*, *sitC*, *sifA*, *sopB*, *spiA*, *cdtB* and *msgA*) in three distinct VT. Different VG are parts of pathogenicity islands in *Salmonella* spp. serovars and can be widely distributed among strains isolated from many animal species (Krawiec et al., 2015; Mokracka et al., 2018). Furthermore, based on the growth on standard media (especially, ChromAgar, MacConkey and SS media), we determined most of the tested reptilian *Salmonella* strains are biochemically atypical compared to clinical strains—their growth looks different compared to clinical samples (Tables 4 and S1). Based on the API20E Test, we identified β‐galactosidase synthesised by 23 *Salmonella* spp. serovars. This is a poorly known aspect of *Salmonella* spp. biology, which can be serovar‐ and pathovar‐specific (Nuccio & Bäumler, 2014), and a result of adaptation to a new host or environment. There is a relevant difference in biochemical properties of gastrointestinal and extraintestinal *Salmonella* spp. serovars. The presence of β‐galactosidase in *Salmonella* spp. is often detected in *S. arizonae* and *S. diarizonae*. These are the so‐called late fermenting subspecies (Lamas et al., 2018; Lenev et al., 2016). Based on the literature, this biochemical property is not connected with the higher virulence of serovars. The role of β‐galactosidase, its variation for *Salmonella* spp. strains and association with environmental factors need further investigation.

## AUTHOR CONTRIBUTIONS

**Aleksandra Pawlak:** Conceptualization (lead); data curation (equal); investigation (lead); methodology (equal); project administration (lead); supervision (equal); writing – original draft (lead); writing – review and editing (lead). **Michał Małaszczuk:** Data curation (equal); investigation (supporting); methodology (supporting); visualization (equal); writing – original draft (supporting); writing – review and editing (supporting). **Mateusz Dróżdż:** Data curation (equal); investigation (supporting); methodology (supporting); visualization (equal); writing – original draft (supporting); writing – review and editing (supporting). **Stanisław Bury:** Conceptualization (supporting); data curation (supporting); formal analysis (supporting); investigation (equal); methodology (supporting); resources (equal); validation (equal); writing – original draft (supporting); writing – review and editing (supporting). **Maciej Kuczkowski:** Data curation (equal); formal analysis (supporting); methodology (supporting); resources (equal); writing – review and editing (supporting). **Katarzyna Morka:** Data curation (equal); formal analysis (supporting); methodology (supporting); validation (equal); writing – review and editing (supporting). **Gabriela Cieniuch:** Data curation (supporting); methodology (supporting). **Agnieszka Korzeniowska‐Kowal:** Data curation (supporting); formal analysis (supporting); funding acquisition (equal); methodology (supporting); resources (equal); software (equal); validation (equal); writing – review and editing (supporting). **Anna Wzorek:** Data curation (supporting); methodology (supporting). **Kamila Korzekwa:** Data curation (supporting); methodology (supporting). **Alina Wieliczko:** Conceptualization (supporting); data curation (supporting); formal analysis (supporting); funding acquisition (equal); project administration (supporting); resources (equal); supervision (supporting); writing – review and editing (supporting). **Mariusz Cichoń:** Conceptualization (supporting); data curation (supporting); funding acquisition (equal); project administration (supporting); resources (equal); writing – review and editing (supporting). **Andrzej Gamian:** Funding acquisition (equal); project administration (supporting); resources (equal); supervision (supporting). **Gabriela Bugla‐Płoskońska:** Conceptualization (supporting); funding acquisition (equal); project administration (supporting); resources (equal); supervision (supporting); writing – review and editing (supporting).

## CONFLICT OF INTEREST STATEMENT

The authors declare no conflict of interest.

## Supporting information


**Table S1.** The juxtaposition of experiments including the growth in selective media and API20E test allowing the identification of *all Salmonella* spp. isolated from *N. natrix* (*n* = 27) and *N. fasciata* (*n* = 3). The percentage of atypical *Salmonella* spp. strains depends on the type of experiment that is taken into account and is shown in Table 3. As compared with positive control sample: clinical reference *Salmonella* spp. isolated from human feaces with salmonellosis, all *Salmonella* spp. isolates are yellow‐red on XLD agar and blue on Simmons agar. Furthermore, all *Salmonella* sp. isolates ferment glucose (GLU) and arabinose (ARA), decarboxylate lysine (LDC) and ornithine (ODC), assimilate citrate (CIT), and produce H_2_S (H_2_S) and NO_2_ (NO_2_), exhibiting the same biochemical features as positive control sample: clinical reference *Salmonella* spp. strain (as shown by Heithoff et al., 2008; Kim et al., 2023; Maddocks et al., 2002; Pao et al., 2005; Svanevik & Lunestad, 2015). All *Salmonella* spp. are incapable of producing urea (URE), do not break down tryptophan (TDA) to indole (IND) and are devoid of cytochrome oxidase—the final enzyme of the respiratory chain in oxygen respiration (OX), which is consistent with biochemical features of clinical reference *Salmonella* spp. strain (as shown by Heithoff et al., 2008; Kim et al., 2023; Maddocks et al., 2002; Pao et al., 2005; Svanevik & Lunestad, 2015). Note all samples should be considered to control samples, which are *Salmonella* reference strains isolated from human feaces suffering from salmonellosis. To determine atypical *Salmonella* spp we suggest searching for ‘–’ cell.

## Data Availability

Data available on request from the authors.

## References

[emi413287-bib-0001] Abd El‐Aziz, N.K. , Tartor, Y.H. , Gharieb, R.M.A. , Erfan, A.M. , Khalifa, E. , Said, M.A. et al. (2021) Extensive drug‐resistant *Salmonella enterica* isolated from poultry and humans: prevalence and molecular determinants behind the co‐resistance to ciprofloxacin and tigecycline. Frontiers in Microbiology, 25(12), 738784. 10.3389/fmicb.2021.738784 PMC8660588 34899627

[emi413287-bib-0002] Alix, E. , Miki, T. , Felix, C. , Rang, C. , Figueroa‐Bossi, N. , Demettre, E. et al. (2008) Interplay between MgtC and PagC in *Salmonella enterica* serovar typhimurium. Microbial Pathogenesis, 45(3), 236–240. 18620040 10.1016/j.micpath.2008.06.001

[emi413287-bib-0003] Baranzelli, A. , Loïez, C. , Bervar, J.F. , Scherpereel, A. & Wallet, F. (2017) The snake raiser lung: an unusual cause of *Salmonella enterica* subspecies *arizonae* pneumonia. Médecine et Maladies Infectieuses, 47(6), 424–425. 28602385 10.1016/j.medmal.2017.05.001

[emi413287-bib-0004] Bauwens, L. , Vercammen, F. , Bertrand, S. , Collard, J.M. & De Ceuster, S. (2006) Isolation of *Salmonella* from environmental samples collected in the reptile departments of Antwerp Zoo using different selective methods. Journal of Applied Microbiology, 101(2), 284–289. 16882135 10.1111/j.1365-2672.2006.02977.x

[emi413287-bib-0005] Bertelloni, F. , Chemaly, M. , Cerri, D. , Le Gall, F. & Ebani, V.V. (2016) *Salmonella* infection in healthy pet reptiles: bacteriological isolation and study of some pathogenic characters. Acta Microbiologica et Immunologica Hungarica, 63(2), 203–216. 27352973 10.1556/030.63.2016.2.5

[emi413287-bib-0006] Bertrand, S. , Rimhanen‐Finne, R. , Weill, F.X. , Rabsch, W. , Thornton, L. , Perevoscikovs, J. et al. (2008) *Salmonella* infections associated with reptiles: the current situation in Europe. Euro Surveillance, 13(24), 18902. 18761944

[emi413287-bib-0007] Bjelland, A.M. , Sandvik, L.M. , Skarstein, M.M. , Svendal, L. & Debenham, J.J. (2020) Prevalence of *Salmonella* serovars isolated from reptiles in Norwegian zoos. Acta Veterinaria Scandinavica, 62(1), 3. 31918736 10.1186/s13028-020-0502-0 PMC6953243

[emi413287-bib-0008] Boadi, S. , Wren, M.W. & Morris‐Jones, S. (2010) Selective testing of β‐galactosidase activity in the laboratory identification of *Salmonella* and *Shigella* species. Journal of Clinical Pathology, 63(12), 1101–1104. 20924038 10.1136/jcp.2010.079723

[emi413287-bib-0009] Bošnjak, I. , Zdravković, N. , Čolović, S. , Ranđelović, S. , Galić, N. , Radojiĉić, M. et al. (2016) Neglected zoonosis: the prevalence of *Salmonella* spp. in pet reptiles in Serbia. Vojnosanitetski Pregled, 73(10), 980–982. 29328567 10.2298/VSP160809222B

[emi413287-bib-0010] Bugla‐Płoskońska, G. , Kiersnowski, A. , Futoma‐Kołoch, B. & Doroszkiewicz, W. (2009) Killing of gram‐negative bacteria with normal human serum and normal bovine serum: use of lysozyme and complement proteins in the death of salmonella strains O48. Microbial Ecology, 58(2), 276–289. 19294463 10.1007/s00248-009-9503-2

[emi413287-bib-0011] Cain, C.R. , Tyre, D. & Ferraro, D. (2009) Incidence of *Salmonella* on reptiles in the pet trade. RURALS: Review of Undergraduate Research in Agricultural and Life Sciences, 4(1), 1.

[emi413287-bib-0013] Centers for Disease Control and Prevention (CDC) . (2018) One health basis . Available at: https://www.cdc.gov/onehealth/basics/index.html [Accessed 15th May 2023].

[emi413287-bib-0014] Centers for Disease Control and Prevention (CDC) . (2020) Salmonella infections linked to pet turtles . Available at: https://www.cdc.gov/salmonella/oranienburg-10-19/index.html [Accessed 15th May 2023].

[emi413287-bib-0015] Chetri, S. , Bhowmik, D. , Paul, D. , Pandey, P. , Chanda, D.D. , Chakravarty, A. et al. (2019) AcrAB‐TolC efflux pump system plays a role in carbapenem non‐susceptibility in *Escherichia coli* . BMC Microbiology, 19, 210. 31488061 10.1186/s12866-019-1589-1 PMC6727511

[emi413287-bib-0016] Corrente, M. , Madio, A. , Friedrich, K.G. , Greco, G. , Desario, C. , Tagliabue, S. et al. (2004) Isolation of *Salmonella* strains from reptile faeces and comparison of different culture media. Journal of Applied Microbiology, 96(4), 709–715. 15012809 10.1111/j.1365-2672.2004.02186.x

[emi413287-bib-0017] Cota, J.B. , Carvalho, A.C. , Dias, I. , Reisinho, A. , Bernardo, F. & Oliveira, M. (2021) *Salmonella* spp. in pet reptiles in Portugal: prevalence and chlorhexidine gluconate antimicrobial efficacy. Antibiotics, 10(3), 324. 33808891 10.3390/antibiotics10030324 PMC8003820

[emi413287-bib-0018] Cristina, R.T. , Kocsis, R. , Dégi, J. , Muselin, F. , Dumitrescu, E. , Tirziu, E. et al. (2022) Pathology and prevalence of antibiotic‐resistant bacteria: a study of 398 pet reptiles. Animals, 12(10), 1279. 35625125 10.3390/ani12101279 PMC9137941

[emi413287-bib-0019] Damborg, P. , Broens, E.M. , Chomel, B.B. , Guenther, S. , Pasmans, F. , Wagenaar, J.A. et al. (2016) Bacterial zoonoses transmitted by household pets: state‐of‐the‐art and future perspectives for targeted research and policy actions. Journal of Comparative Pathology, 155(1), S27–S40. 25958184 10.1016/j.jcpa.2015.03.004

[emi413287-bib-0021] Dudek, B. , Książczyk, M. , Krzyżewska, E. , Rogala, K. , Kuczkowski, M. , Woźniak‐Biel, A. et al. (2019) Comparison of the phylogenetic analysis of PFGE profiles and the characteristic of virulence genes in clinical and reptile associated *Salmonella* strains. BMC Veterinary Research, 15(312), 312. 31477105 10.1186/s12917-019-2019-1 PMC6721270

[emi413287-bib-0022] Ebani, V.V. , Cerri, D. , Fratini, F. , Meille, N. , Valentini, P. & Andreani, E. (2005) *Salmonella enterica* isolates from faeces of domestic reptiles and a study of their antimicrobial in vitro sensitivity. Research in Veterinary Science, 78(2), 117–121. 15563917 10.1016/j.rvsc.2004.08.002

[emi413287-bib-0023] European Food Safety Authority (EFSA) & European Centre for Disease Prevention and Control (ECDC) . (2019) The European Union summary report on antimicrobial resistance in zoonotic and indicator bacteria from humans, animals and food in 2017. European Food Safety Authority Journal, 17(2), e05598. 32626224 10.2903/j.efsa.2019.5598 PMC7009238

[emi413287-bib-0024] European Committee on Antimicrobial Susceptibility Testing . (2021) *Breakpoint tables for interpretation of MICs and zone diameters*. Version 11.0, 2021. Available at: http://www.eucast.org [Accessed 15th May 2023].

[emi413287-bib-0025] European Food Safety Authority . (2019) The European Union one health 2018. Zoonoses Report, 17(12), 5926. 10.2903/j.efsa.2019.5926 PMC7055727 32626211

[emi413287-bib-0026] Gambino‐Shirley, K. , Stevenson, L. , Concepción‐Acevedo, J. , Trees, E. , Wagner, D. , Whitlock, L. et al. (2018) Flea market finds and global exports: four multistate outbreaks of human *Salmonella* infections linked to small turtles, USA—2015. Zoonoses and Public Health, 65(5), 560–568. 29577654 10.1111/zph.12466

[emi413287-bib-0027] Gavrilovici, C. , Pânzaru, C.V. , Cozma, S. , Mârţu, C. , Lupu, V.V. , Ignat, A. et al. (2017) Message from a turtle: otitis with *Salmonella arizonae* in children case report. Medicine, 96(44), e8455. 29095293 10.1097/MD.0000000000008455 PMC5682812

[emi413287-bib-0028] Geue, L. & Löschner, U. (2002) Salmonella enterica in reptiles of German and Austrian origin. Veterinary Microbiology, 84(1–2), 79–91. 11731161 10.1016/s0378-1135(01)00437-0

[emi413287-bib-0029] Gunn, J.S. , Alpuche‐Aranda, C.M. , Loomis, W.P. , Belden, W.J. & Miller, S. (1995) Characterization of the *Salmonella* typhimurium pagC/pagD chromosomal. The Region, 177(17), 5040–5047. 10.1128/jb.177.17.5040-5047.1995 PMC177282 7665482

[emi413287-bib-0030] Hacioglu, N. , Gul, C. & Tosunoglu, M. (2015) Bacteriological screening and antibiotic–heavy metal resistance profile of the bacteria isolated from some amphibian and reptile species of the Biga stream in Turkey. International Journal of Environmental and Ecological Engineering, 9(4), 422–426.

[emi413287-bib-0031] Harris, J.R. , Neil, K.P. , Behravesh, B.C. , Sotir, M.J. & Angulo, F.J. (2010) Recent multistate outbreaks of human salmonella infections acquired from turtles: a continuing public health challenge. Clinical Infectious Diseases, 50(4), 554–559. 20085463 10.1086/649932

[emi413287-bib-0032] Heithoff, D.M. , Shimp, W.R. , Lau, P.W. , Badie, G. , Enioutina, E.Y. , Daynes, R.A. et al. (2008) Human *Salmonella* clinical isolates distinct from those of animal origin. Applied and Environmental Microbiology, 74(6), 1757–1766. 18245251 10.1128/AEM.02740-07 PMC2268321

[emi413287-bib-0033] Hibbitts, T.J. & Fitzgerald, L.A. (2005) Morphological and ecological convergence in two natricine snakes. Biological Journal of the Linnean Society, 85(3), 363–371.

[emi413287-bib-0034] Holmes, E.C. , Goldstein, S.A. , Rasmussen, A.L. , Robertson, D.L. , Crits‐Christoph, A. , Wertheim, J.O. et al. (2021) The origins of SARS‐CoV‐2: a critical revive. Cell, 184(19), 4848–4856. 34480864 10.1016/j.cell.2021.08.017 PMC8373617

[emi413287-bib-0035] Horvath, L. , Kraft, M. , Fostiropoulos, K. , Falkowski, A. & Tarr, P.E. (2016) *Salmonella enterica* subspecies diarizonae maxillary sinusitis in a snake handler: first report. Open Forum Infectious Diseases, 3(2):ofw066. 27186588 10.1093/ofid/ofw066 PMC4866548

[emi413287-bib-0036] Hunter, P.R. & Gaston, M.A. (1998) Numerical index of the discriminatory ability of typing systems: an application of Simpson's index of diversity. Journal of Clinical Microbiology, 26(11), 2465–2466. 10.1128/jcm.26.11.2465-2466.1988 PMC266921 3069867

[emi413287-bib-0037] Hydeskov, H.B. , Guardabassi, L. , Aalbaek, B. , Olsen, K.E. , Nielsen, S.S. & Bertelsen, M.F. (2013) *Salmonella* prevalence among reptiles in a zoo education setting. Zoonoses and Public Health, 60(4), 291–295. 22835051 10.1111/j.1863-2378.2012.01521.x

[emi413287-bib-0038] Jang, Y.H. , Lee, S.J. , Lim, J.G. , Lee, H.S. , Kim, T.J. , Park, J.H. et al. (2008) The rate of *Salmonella* spp. infection in zoo animals at Seoul Grand Park Korea. Journal of Veterinary Science, 9(2), 177–181. 18487939 10.4142/jvs.2008.9.2.177 PMC2839095

[emi413287-bib-0039] Kiebler, C.A. , Bottichio, L. , Simmons, L. , Basler, C. , Klos, R. , Gurfield, N. et al. (2020) Outbreak of human infections with uncommon salmonella serotypes linked to pet bearded dragons, 2012‐2014. Zoonoses and Public Health, 67(4), 425–434. 32304287 10.1111/zph.12701 PMC11325769

[emi413287-bib-0040] Kim, H.J. , Jung, Y. , Kim, M.J. & Kim, H.Y. (2023) Novel heptaplex PCR‐based diagnostics for enteric fever caused by typhoidal *Salmonella* serovars and its applicability in clinical blood culture. Journal of Microbiology and Biotechnology, 33(11), 1457–1466. 37674393 10.4014/jmb.2307.07031 PMC10699274

[emi413287-bib-0041] Kim, W.Y. , Moon, J.Y. , Huh, J.W. , Choi, S.H. , Lim, C.M. , Koh, Y. et al. (2016) Comparable efficacy of tigecycline versus colistin therapy for multidrug‐resistant and extensively drug‐resistant *Acinetobacter baumannii* pneumonia in critically ill patients. PLoS One, 11(3), e0150642. 26934182 10.1371/journal.pone.0150642 PMC4775052

[emi413287-bib-0042] Kontou, M. , Pournaras, S. , Kristo, I. , Ikonomidis, A. , Maniatis, A.N. & Stathopoulos, C. (2007) Molecular cloning and biochemical characterization of VIM‐12, a novel hybrid VIM‐1/VIM‐2 metallo‐beta‐lactamase from a *Klebsiella pneumoniae* clinical isolate, reveal atypical substrate specificity. Biochemistry, 46(45), 13170–13178. 17944487 10.1021/bi701258w

[emi413287-bib-0043] Krautwald‐Junghanns, M.E. , Stenkat, J. , Szabo, I. , Ortlieb, F. , Blindow, I. , Neul, A.K. et al. (2013) Characterization of Salmonella isolated from captive and free‐living snakes in Germany. Berliner und Münchener Tierärztliche Wochenschrift, 126(5–6), 209–215. 23758035

[emi413287-bib-0044] Krawiec, M. , Kuczkowski, M. , Kruszewicz, A.G. & Wieliczko, A. (2015) Prevalence and genetic characteristics of *Salmonella* in free‐living birds in Poland. BCM Veterinary Research, 11, 15. 10.1186/s12917-015-0332-x PMC4316766 25636375

[emi413287-bib-0045] Krzyżewska‐Dudek, E. , Kotimaa, J. , Kapczyńska, K. , Rybka, J. & Meri, S. (2022) Lipopolysaccharides and outer membrane proteins as main structures involved in complement evasion strategies of non‐typhoidal *Salmonella* strains. Molecular Immunology, 150, 67–77. 35998438 10.1016/j.molimm.2022.08.009

[emi413287-bib-0046] Kurtz, J.R. , Goggins, J.A. & McLachlan, J.B. (2017) *Salmonella* infection: interplay between the bacteria and host immune system. Immunology Letters, 190, 42–50. 28720334 10.1016/j.imlet.2017.07.006 PMC5918639

[emi413287-bib-0047] Lamas, A. , Miranda, J.M. , Regal, P. , Vázquez, B. , Franco, C.M. & Cepeda, A. (2018) A comprehensive review of non‐enterica subspecies of salmonella enterica. Microbiological Research, 206, 60–73. 29146261 10.1016/j.micres.2017.09.010

[emi413287-bib-0048] Lee, K. , Iwata, T. , Shimizu, M. , Taniguchi, T. , Nakadai, A. , Hirota, Y. et al. (2009) A novel multiplex PCR assay for *Salmonella* subspecies identification. Journal of Applied Microbiology, 107(3), 805–811. 19486419 10.1111/j.1365-2672.2009.04263.x

[emi413287-bib-0049] Lenev, S. , Laishevtsev, A. , Pimenov, N. , Semykin, V. , Pigorev, I. , Eremenko, V. et al. (2016) Improvement of allocation and identification of *Salmonella entericabacteria* of arizonae subspecies. International Journal of Pharmaceutical Research & Allied Sciences, 5(2), 342–348.

[emi413287-bib-0050] Lilic, M. , Quezada, C.M. & Stebbins, C.E. (2010) A conserved domain in type III secretion links the cytoplasmic domain of InvA to elements of the basal body. Acta Crystallographica. Section D, Biological Crystallography, 66(6), 709–713. 20516623 10.1107/S0907444910010796 PMC2879356

[emi413287-bib-0051] Lukač, M. , Horvatek Tomić, D. , Mandac, Z. , Mihoković, S. & Prukner‐Radovčić, E. (2017) Oral and cloacal aerobic bacterial and fungal flora of free‐living four‐lined snakes (*Elaphe quatuorlineata*) from Croatia. Veterinarski Arhiv, 87, 351–361.

[emi413287-bib-0052] Maddocks, S. , Olma, T. & Chen, S. (2002) Comparison of CHROMagar Salmonella medium and xylose‐lysine desoxycholate and Salmonella‐Shigella agars for isolation of Salmonella strains from stool samples. Journal of Clinical Microbiology, 40(8), 2999–3003. 12149365 10.1128/JCM.40.8.2999-3003.2002 PMC120614

[emi413287-bib-0053] Magouras, I. , Brookes, V.J. , Jori, F. , Martin, A. , Pfeiffer, D.U. & Dürr, S. (2020) Emerging zoonotic diseases: should we rethink the animal–human Interface? Frontiers in Veterinary Science, 7, 582743. 33195602 10.3389/fvets.2020.582743 PMC7642492

[emi413287-bib-0054] Marin, C. , Lorenzo‐Rebenaque, L. , Laso, O. , Villora‐Gonzalez, J. & Vega, S. (2021) Pet reptiles: a potential source of transmission of multidrug‐resistant *Salmonella* . Frontiers in Veterinary Science, 7, 613718. 33490138 10.3389/fvets.2020.613718 PMC7815585

[emi413287-bib-0055] Meervenne, E.V. , Botteldoorn, N. , Lokietek, S. , Vatlet, M. , Cupa, A. , Naranjo, M. et al. (2009) Turtle‐associated *Salmonella* septicaemia and meningitides in a 2‐month‐old baby. Journal of Medical Microbiology, 58, 1379–1381. 19528160 10.1099/jmm.0.012146-0

[emi413287-bib-0056] Mermin, J. , Hoar, B. & Angulo, F.J. (1997) Iguanas and *Salmonella* marina infection in children: a reflection of the increasing incidence of reptile‐associated salmonellosis in the United States. Pediatrics, 99(3), 399–402. 9041295 10.1542/peds.99.3.399

[emi413287-bib-0057] Meyer Sauteur, P.M. , Relly, C. , Hug, M. , Wittenbrink, M.M. & Berger, C. (2013) Risk factors for invasive reptile‐associated salmonellosis in children. Vector Borne and Zoonotic Diseases, 13(6), 419–421. 23473215 10.1089/vbz.2012.1133

[emi413287-bib-0058] Mokracka, J. , Krzymińska, S. , Ałtunin, D. , Wasyl, D. , Koczura, R. , Dudek, K. et al. (2018) In vitro virulence characteristic of rare serovars of *Salmonella enterica* isolated from sand lizards (*Lacerta agilis* L.). Antonie Van Leeuwenhoek, 111(10), 1863–1870. 29779148 10.1007/s10482-018-1079-8 PMC6153992

[emi413287-bib-0060] Morydz, W. , Okarma, H. & Skórka, P. (2017) Environmental features associated with a presence of red‐eared slider Trachemys scripta elegans in the wild in Poland . Available at: https://ruj.uj.edu.pl/xmlui/handle/item/218026 [Accessed 12 March 2024].

[emi413287-bib-0061] Mourão, J. , Rebelo, A. , Ribeiro, S. , Peixe, L. , Novais, C. & Antunes, P. (2020) Atypical non‐H_2_S‐producting *Salmonella* typhimurium ST3478 strains from chicken meat at processing stage are adapted to diverse stress. Pathogens, 9(9), 701. 32859122 10.3390/pathogens9090701 PMC7557518

[emi413287-bib-0062] Mrozowska, A. & Tyc, Z. (1992) Strains of *Salmonella* with atypical biochemical activity found in diagnostic material. Medycyna Doświadczalna i Mikrobiologia, 44(3–4), 109–117. 1305915

[emi413287-bib-0063] Mughini‐Gras, L. , Heck, M. & Van Pelt, W. (2016) Increase in reptile‐associated human salmonellosis and shift toward adulthood in the age groups at risk, The Netherlands, 1985 to 2014. Euro Surveillance, 21(34), 30324. 27589037 10.2807/1560-7917.ES.2016.21.34.30324 PMC5144934

[emi413287-bib-0064] Nakadai, A. , Kuroki, T. , Kato, Y. , Suzuki, R. , Yamai, S. , Yaginuma, C. et al. (2005) Prevalence of *Salmonella* spp. in pet reptiles in Japan. The Journal of Veterinary Medical Science, 67(1), 97–101. 15699603 10.1292/jvms.67.97

[emi413287-bib-0065] Nishio, M. , Okada, N. , Mik, I.T. , Haneda, T. & Danbara, H. (2005) Identification of the outer‐membrane protein PagC required for the serum resistance phenotype in *Salmonella enterica* serovar choleraesuis. Microbiology, 151(Pt 3), 863–873. 15758232 10.1099/mic.0.27654-0

[emi413287-bib-0066] Nowakiewicz, A. , Ziółkowska, G. , Zięba, P. , Majer Dziedzic, B. , Gnat, S. , Wójcik, M. et al. (2015) Aerobic bacterial microbiota isolated from the cloaca of the European pond turtle (*Emys orbicularis*) in Poland. Journal of Wildlife Diseases, 51(1), 255–259. 25380369 10.7589/2013-07-157

[emi413287-bib-0067] Nuccio, S.P. & Bäumler, A.J. (2014) Comparative analysis of *Salmonella* genomes identifies a metabolic network for escalating growth in the inflamed gut. MBio, 5(2), e00929‐14. 24643865 10.1128/mBio.00929-14 PMC3967523

[emi413287-bib-0068] Ondari, E.M. , Klemm, E.J. , Msefula, C.L. , El Ghany, M.A. , Heath, J.N. , Pickard, D.J. et al. (2019) Rapid transcriptional responses to serum exposure are associated with sensitivity and resistance to antibody‐mediated complement killing in invasive *Salmonella* Typhimurium ST313. Wellcome Open Research, 25(4), 74. 10.12688/wellcomeopenres.15059.1 PMC6560496 31231691

[emi413287-bib-0070] Pao, S. , Patel, D. , Kalantari, A. , Tritschler, J.P. , Wildeus, S. & Sayre, B.L. (2005) Detection of *Salmonella* strains and *Escherichia coli* O157:H7 in feces of small ruminants ant their isolation with various media. Applied and Environmental Microbiology, 71(4), 2158–2161. 15812051 10.1128/AEM.71.4.2158-2161.2005 PMC1082527

[emi413287-bib-0071] Pasmans, F. , Martel, A. , Boyen, F. , Vandekerchove, D. , Wybo, I. , Van Immerseel, F. et al. (2005) Characterization of *Salmonella* isolates from captive lizards. Veterinary Microbiology, 110(3–4), 285–291. 16153787 10.1016/j.vetmic.2005.07.008

[emi413287-bib-0072] Pawlak, A. , Morka, K. , Bury, S. , Antoniewicz, Z. , Wzorek, A. , Cieniuch, G. et al. (2020) Cloacal gram‐negative microbiota in free‐living grass snake *Natrix natrix* from Poland. Current Microbiology, 77(9), 2166–2171. 32424607 10.1007/s00284-020-02021-3 PMC7415037

[emi413287-bib-0073] Pawlak, A. , Rybka, J. , Dudek, B. , Krzyżewska, E. , Rybka, W. , Kędziora, A. et al. (2017) Salmonella O48 serum resistance is connected with the elongation of the lipopolysaccharide O‐antigen containing sialic acid. International Journal of Molecular Sciences, 18(10), 2022. 28934165 10.3390/ijms18102022 PMC5666704

[emi413287-bib-0074] Pees, M. , Rabsch, W. , Plenz, B. , Fruth, A. , Prager, R. , Simon, S. et al. (2013) Evidence for the transmission of *Salmonella* from reptiles to children in Germany, July 2010 to October 2011. Euro Surveillance, 18(46), 20634. 24256890 10.2807/1560-7917.es2013.18.46.20634

[emi413287-bib-0075] Pérez Costa, E. , Molina Gutiérrez, M.A. & Escosa Garcia, L. (2015) Los riesgos del empleo de reptiles como animales de compañía. Revista de Pediatría de Atención Primaria, 17(66), 129–131.

[emi413287-bib-0076] Rossi, O. , Coward, C. , Goh, Y.S. , Claassens, J.W.C. , MacLennan, C.A. , Verbeek, S.J. et al. (2019) The essential role of complement in antibody‐mediated resistance to *Salmonella* . Immunology, 156(1), 69–73. 30179254 10.1111/imm.13000 PMC6283648

[emi413287-bib-0077] Russo, T.P. , Varriale, L. , Borrelli, L. , Pace, A. , Latronico, M. , Menna, L.F. et al. (2018) *Salmonella* serotypes isolated in geckos kept in seven collections in southern Italy. The Journal of Small Animal Practice, 59(5), 294–297. 29315571 10.1111/jsap.12808

[emi413287-bib-0078] Schmidt, V. , Mock, R. , Burgkhardt, E. , Junghanns, A. , Ortlieb, F. , Szabo, I. et al. (2014) Cloacal aerobic bacterial flora and absence of viruses in free‐living slow worms (*Anguis fragilis*), grass snakes (*Natrix natrix*) and European adders (*Vipera berus*) from Germany. EcoHealth, 11(4), 571–580. 24866333 10.1007/s10393-014-0947-6

[emi413287-bib-0079] Schneider, L. , Ehlinger, M. , Stanchina, C. , Giacomelli, M.C. , Gicquel, P. , Karger, C. et al. (2009) *Salmonella enterica* subsp. arizonae bone and joints sepsis. A case report and literature review. Orthopaedics & Traumatology, Surgery & Research, 95(3), 237–242. 10.1016/j.otsr.2008.09.010 19395336

[emi413287-bib-0080] Skoczylas, R. (1970) Influence of temperature on gastric digestion in the grass snake *Natrix natrix* L. Comparative Biochemistry and Physiology, 33, 793–804.

[emi413287-bib-0081] Skyberg, J.A. , Logue, C.M. & Nolan, L.K. (2006) Virulence genotyping of *Salmonella* spp. with multiplex PCR. Avian Diseases, 50(1), 77–81. 16617986 10.1637/7417.1

[emi413287-bib-0082] Stein, M.A. , Leung, K.Y. , Zwick, M. , Garcia‐del Portillo, F. & Finlay, B.B. (1996) Identification of a Salmonella virulence gene required for formation of filamentous structures containing lysosomal membrane glycoproteins within epithelial cells. Molecular Microbiology, 20(1), 151–164. 8861213 10.1111/j.1365-2958.1996.tb02497.x

[emi413287-bib-0083] Suzuki, A. , Tanaka, T. , Ohba, K. , Ito, N. , Sakai, Y. , Kaneko, A. et al. (2017) Purulent pericarditis with *Salmonella enterica* subspecies arizona in a patient with type 2 diabetes mellitus. Internal Medicine, 56(16), 2171–2174. 28781305 10.2169/internalmedicine.8293-16 PMC5596279

[emi413287-bib-0084] Svanevik, C.S. & Lunestad, B.T. (2015) Microbiological water examination during laboratory courses generates new knowledge for students, scientists and the government. FEMS Microbiology Letters, 362(20), 151. 10.1093/femsle/fnv151 26337153

[emi413287-bib-0085] Townsend, M.L. , Pound, M.W. & Drew, R.H. (2007) Tigecycline in the treatment of complicated intra‐abdominal and complicated skin and skin structure infections. Therapeutics and Clinical Risk Management, 3(6), 1059–1070. 18516315 PMC2387284

[emi413287-bib-0086] Vora, N.M. , Smith, K.M. , Machalaba, C.C. & Karesh, W.B. (2012) Reptile‐ and amphibian‐associated salmonellosis in childcare centres, United States. Emerging Infectious Diseases, 18(12), 2092–2094. 23171538 10.3201/eid1812.120784 PMC3557882

[emi413287-bib-0087] Wang, Z. , Jiang, Y. , Xu, H. , Jiao, X. , Wang, J. & Li, Q. (2023) Poultry production as the main reservoir of ciprofloxacin‐ and tigecycline‐resistant extended‐spectrum β‐lactamase (ESBL)‐producing *Salmonella enterica* serovar Kentucky ST198.2‐2 causing human infections in China. Applied and Environmental Microbiology, 89(9), e0094423. 37610223 10.1128/aem.00944-23 PMC10537671

[emi413287-bib-0088] Whiley, H. , Gardner, M.G. & Ross, K. (2017) A review of *Salmonella* and squamates (lizards, snakes and amphibians): implications for public health. Pathogens, 6(3), 38. 28829352 10.3390/pathogens6030038 PMC5617995

[emi413287-bib-0089] Wikström, V.O. , Fernström, L.L. , Melin, L. & Boqvist, S. (2014) *Salmonella* isolated from individual reptiles and environmental samples from terraria in private households in Sweden. Acta Veterinaria Scandinavica, 56(1), 7. 24461167 10.1186/1751-0147-56-7 PMC3922756

[emi413287-bib-0090] World Health Organization (WHO) . (2021) Zoonotic disease: emerging public health threats in the region . Available at: http://www.emro.who.int/about-who/rc61/zoonotic-diseases.html [Accessed 15th May 2023].

[emi413287-bib-0091] Xia, Y. , Li, H. & Shen, Y. (2020) Antimicrobial drug resistance in *Salmonella enteritidis* isolated from edible snakes with pneumonia and its pathogenicity in chickens. Frontiers in Veterinary Science, 7, 463. 32851038 10.3389/fvets.2020.00463 PMC7417342

[emi413287-bib-0092] Yaghoubi, S. , Zekiy, A.O. , Krutova, M. , Gholami, M. , Kouhsari, E. , Sholeh, M. et al. (2022) Tigecycline antibacterial activity, clinical effectiveness, and mechanisms and epidemiology of resistance: narrative review. European Journal of Clinical Microbiology & Infectious Diseases, 41(7), 1003–1022. 33403565 10.1007/s10096-020-04121-1 PMC7785128

[emi413287-bib-0093] Zając, M. , Wasyl, D. , Hoszowski, A. , Le Hello, S. & Szulowski, K. (2013) Genetic lineages of *Salmonella enterica* serovar Kentucky spreading in pet reptiles. Veterinary Microbiology, 166(3–4), 686–689. 23962467 10.1016/j.vetmic.2013.07.023

[emi413287-bib-0094] Zając, M. , Wasyl, D. , Różycki, M. , Bilska‐Zając, E. , Fafiński, Z. , Iwaniak, W. et al. (2016) Free‐living snakes as a source and possible vector of *Salmonella* spp. and parasites. European Journal of Wildlife Research, 62, 161–166.

[emi413287-bib-0095] Zhang, Z. , Tian, X. & Shi, C. (2022) Global spread of MCR‐producing *Salmonella enterica* isolates. Antibiotics, 11(8), 998. 35892388 10.3390/antibiotics11080998 PMC9330719

