# Supplementary material for: Virulence factors of Salmonella spp. isolated from free‐living grass snakes Natrix natrix
Source: Environ Microbiol Rep. 2024 Jul 8;16(4):e13287. doi: 10.1111/1758-2229.13287 (PMC11231047; doi:10.1111/1758-2229.13287)
Supplement: Supplementary file 1 — Table S1. The juxtaposition of experiments including the growth in selective media and API20E test allowing the identification of all Salmonella spp. isolated from N. natrix (n = 27) and N. fasciata (n = 3). The percentage of atypical Salmonella spp. strains depends on the type of experiment that is taken into account and is shown in Table 3. As compared with positive control sample: clinical reference Salmonella spp. isolated from human feaces with salmonellosis, all Salmonella spp. isolates are yellow‐red on XLD agar and blue on Simmons agar. Furthermore, all Salmonella sp. isolates ferment glucose (GLU) and arabinose (ARA), decarboxylate lysine (LDC) and ornithine (ODC), assimilate citrate (CIT), and produce H2S (H2S) and NO2 (NO2), exhibiting the same biochemical features as positive control sample: clinical reference Salmonella spp. strain (as shown by Heithoff et al., 2008; Kim et al., 2023; Maddocks et al., 2002; Pao et al., 2005; Svanevik & Lunestad, 2015). All Salmonella spp. are incapable of producing urea (URE), do not break down tryptophan (TDA) to indole (IND) and are devoid of cytochrome oxidase—the final enzyme of the respiratory chain in oxygen respiration (OX), which is consistent with biochemical features of clinical reference Salmonella spp. strain (as shown by Heithoff et al., 2008; Kim et al., 2023; Maddocks et al., 2002; Pao et al., 2005; Svanevik & Lunestad, 2015). Note all samples should be considered to control samples, which are Salmonella reference strains isolated from human feaces suffering from salmonellosis. To determine atypical Salmonella spp we suggest searching for ‘–’ cell. [file EMI4-16-e13287-s001.docx]

**Table S1: The juxtaposition of experiments including the growth in selective media and API20E test allowing the identification of all *Salmonella* spp. isolated from *N. natrix* (n=27) and *N. fasciata* (n=3).** The percentage of atypical *Salmonella* spp. strains depends on the type of experiment that is taken into account and is shown in Table 3. As compared with positive control sample: clinical reference *Salmonella* spp. isolated from human feaces with salmonellosis, all *Salmonella* spp. isolates are yellow-red on XLD agar and blue on Simmons agar. Furthermore, all *Salmonella* sp. isolates ferment glucose (GLU) and arabinose (ARA), decarboxylate lysine (LDC) and ornithine (ODC), assimilate citrate (CIT), and produce H_2_S (H_2_S) and NO_2_ (NO_2_)_,_ exhibiting the same biochemical features as positive control sample: clinical reference *Salmonella* spp. strain (as shown by Maddocks et al., 2002, Pao et al., 2005, Svansevik et al., 2015, Heithoff et al., 2008, Kim et al., 2023). All *Salmonella* spp. are incapable of producing urea (URE), do not break down tryptophan (TDA) to indole (IND) and are devoid of cytochrome oxidase - the final enzyme of the respiratory chain in oxygen respiration (OX), which is consistent with biochemical features of clinical reference *Salmonella* spp. strain (as shown by Maddocks et al., 2002, Pao et al., 2005, Svansevik et al., 2015, Heithoff et al., 2008, Kim et al., 2023). Note all samples should be considered to control samples, which are *Salmonella* reference strains isolated from human feaces suffering from salmonellosis. To determine atypical *Salmonella* spp we suggest searching for “–“ cell. .

| **Isolate** | **Culture media** | | | | | **API 20E** | | | | | | | | | | | |
| --- | --- | --- | --- | --- | --- | --- | --- | --- | --- | --- | --- | --- | --- | --- | --- | --- | --- |
|  | **SS**  **Agar** | **MacConkey**  **Agar** | **ChromAgar** | **XLD Agar** | **Simmons Agar** | **ONPG** | **GLU** | **ARA** | **LDC** | **ODC** | **CIT** | **H2S** | **URE** | **TDA** | **IND** | **OX** | **NO2** |
| **Clinical sample** | + | + | + | + | + | + | + | + | + | + | + | + | + | + | + | + | + |
| **NF 9.2** | + | + | + | + | + | - | + | + | + | + | + | + | + | + | + | + | + |
| **NF 9.4** | + | + | + | + | + | - | + | + | + | + | + | + | + | + | + | + | + |
| **NF 9.5** | + | + | + | + | + | - | + | + | + | + | + | + | + | + | + | + | + |
| **NN 1.1** | - | - | + | + | + | - | + | + | + | + | + | + | + | + | + | + | + |
| **NN 1.2** | - | - | + | + | + | - | + | + | + | + | + | + | + | + | + | + | + |
| **NN 1.3** | + | - | + | + | + | - | + | + | + | + | + | + | + | + | + | + | + |
| **NN 8.1** | + | - | + | + | + | - | + | + | + | + | + | + | + | + | + | + | + |
| **NN 9.2** | + | - | + | + | + | - | + | + | + | + | + | + | + | + | + | + | + |
| **NN 11.1** | + | - | + | + | + | - | + | + | + | + | + | + | + | + | + | + | + |
| **NN 12.2** | + | + | **-** | + | + | + | + | + | + | + | + | + | + | + | + | + | + |
| **NN 13.1** | + | + | + | + | + | - | + | + | + | + | + | + | + | + | + | + | + |
| **NN 13.3** | + | + | + | + | + | - | + | + | + | + | + | + | + | + | + | + | + |
| **NN 14.3** | + | - | + | + | + | - | + | + | + | + | + | + | + | + | + | + | + |
| **NN 14.4** | + | + | + | + | + | - | + | + | + | + | + | + | + | + | + | + | + |
| **II NN 4.7** | - | + | + | + | + | - | + | + | + | + | + | + | + | + | + | + | + |
| **II NN 6.1** | + | + | **-** | + | + | + | + | + | + | + | + | + | + | + | + | + | + |
| **III NN 14.3** | - | - | + | + | + | - | + | + | + | + | + | + | + | + | + | + | + |
| **III NN 14.5** | + | - | + | + | + | - | + | + | + | + | + | + | + | + | + | + | + |
| **III NN14.6** | + | - | + | + | + | - | + | + | + | + | + | + | + | + | + | + | + |
| **39.1K** | + | + | + | + | + | - | + | + | + | + | + | + | + | + | + | + | + |
| **1.2S** | + | + | + | + | + | + | + | + | + | + | + | + | + | + | + | + | + |
| **4.1S** | - | + | **-** | + | + | + | + | + | + | + | + | + | + | + | + | + | + |
| **7.1S** | - | + | + | + | + | - | + | + | + | + | + | + | + | + | + | + | + |
| **11S** | - | + | + | + | + | - | + | + | + | + | + | + | + | + | + | + | + |
| **13.3S** | - | + | + | + | + | - | + | + | + | + | + | + | + | + | + | + | + |
| **28.1S** | + | - | + | + | + | + | + | + | + | + | + | + | + | + | + | + | + |
| **3.1L** | + | - | + | + | + | - | + | + | + | + | + | + | + | + | + | + | + |
| **24.2L** | + | + | + | + | + | - | + | + | + | + | + | + | + | + | + | + | + |
| **II 16.2K** | - | + | + | + | + | + | + | + | + | + | + | + | + | + | + | + | + |
| **II 4.1S** | - | + | **-** | + | + | + | + | + | + | + | + | + | + | + | + | + | + |

*+* - result of the growth of *Salmonella* spp. on the selective media or biochemical features based on API20E test, which are **consistent** with a control sample: clinical reference *Salmonella* spp.

­- - result of the growth of *Salmonella* spp. on the selective media or biochemical features based on API20E test, which are **not consistent** with a control sample: clinical reference *Salmonella* spp
